# Targeting delivery of miR-146a via IMTP modified milk exosomes exerted cardioprotective effects by inhibiting NF-κB signaling pathway after myocardial ischemia-reperfusion injury

**DOI:** 10.1186/s12951-024-02631-0

**Published:** 2024-07-01

**Authors:** Wan-ting Meng, Jing Zhu, Ya-chao Wang, Chang-le Shao, Xiu-ya Li, Ping-ping Lu, Meng-ying Huang, Fang-fang Mou, Hai-dong Guo, Guang Ji

**Affiliations:** 1 Academy of Integrative Medicine, Shanghai University of Traditional Chinese Medicine, Shanghai, 201203 China; 2 Institute of Digestive Diseases, Longhua Hospital, China-Canada Center of Research for Digestive Diseases (ccCRDD), Shanghai University of Traditional Chinese Medicine, Shanghai, 200032 China

**Keywords:** Myocardial ischemia-reperfusion injury, Milk exosome, microRNA-146a, Targeting delivery, Inflammatory factors, NF-κB signaling pathway

## Abstract

Reperfusion therapy is critical for saving heart muscle after myocardial infarction, but the process of restoring blood flow can itself exacerbate injury to the myocardium. This phenomenon is known as myocardial ischemia-reperfusion injury (MIRI), which includes oxidative stress, inflammation, and further cell death. microRNA-146a (miR-146a) is known to play a significant role in regulating the immune response and inflammation, and has been studied for its potential impact on the improvement of heart function after myocardial injury. However, the delivery of miR-146a to the heart in a specific and efficient manner remains a challenge as extracellular RNAs are unstable and rapidly degraded. Milk exosomes (MEs) have been proposed as ideal delivery platform for miRNA-based therapy as they can protect miRNAs from RNase degradation. In this study, the effects of miR-146a containing MEs (MEs-miR-146a) on improvement of cardiac function were examined in a rat model of MIRI. To enhance the targeting delivery of MEs-miR-146a to the site of myocardial injury, the ischemic myocardium-targeted peptide IMTP was modified onto the surfaces, and whether the modified MEs-miR-146a could exert a better therapeutic role was examined by echocardiography, myocardial injury indicators and the levels of inflammatory factors. Furthermore, the expressions of miR-146a mediated NF-κB signaling pathway-related proteins were detected by western blotting and qRT-PCR to further elucidate its mechanisms. MiR-146 mimics were successfully loaded into the MEs by electroporation at a square wave 1000 V voltage and 0.1 ms pulse duration. MEs-miR-146a can be up-taken by cardiomyocytes and protected the cells from oxygen glucose deprivation/reperfusion induced damage in vitro. Oral administration of MEs-miR-146a decreased myocardial tissue apoptosis and the expression of inflammatory factors and improved cardiac function after MIRI. The miR-146a level in myocardium tissues was significantly increased after the administration IMTP modified MEs-miR-146a, which was higher than that of the MEs-miR-146a group. In addition, intravenous injection of IMTP modified MEs-miR-146a enhanced the targeting to heart, improved cardiac function, reduced myocardial tissue apoptosis and suppressed inflammation after MIRI, which was more effective than the MEs-miR-146a treatment. Moreover, IMTP modified MEs-miR-146a reduced the protein levels of IRAK1, TRAF6 and p-p65. Therefore, IMTP modified MEs-miR-146a exerted their anti-inflammatory effect by inhibiting the IRAK1/TRAF6/NF-κB signaling pathway. Taken together, our findings suggested miR-146a containing MEs may be a promising strategy for the treatment of MIRI with better outcome after modification with ischemic myocardium-targeted peptide, which was expected to be applied in clinical practice in future.

**Supplementary Information:**

The online version contains supplementary material available at 10.1186/s12951-024-02631-0.

## Introduction

Myocardial ischemia-reperfusion injury (MIRI) is a complex phenomenon that can occur in ≥ 60% of patients after procedures like angioplasty or coronary artery bypass surgery, or during the treatment of a heart attack [1]. When the blood supply is restored after a period of ischemia, it can exacerbate the damage to the heart tissue [2, 3]. This is due to a sudden surge of blood causing an inflammatory response and oxidative stress in the previously oxygen-deprived tissues. The mechanisms contributing to MIRI normally include oxidative stress, calcium overload, inflammation and endothelial dysfunction [4, 5]. Due to the absence of effective clinical interventions, the clinical concern about MIRI has attracted widespread attention.

MicroRNA (miRNA) are endogenous small non-coding RNA molecules, which as an essential regulator of gene expression post-transcriptionally [6]. miRNA plays a crucial role in the pathogenesis and progression of MIRI [7, 8]. Strategies to upregulate protective miRNAs or inhibit detrimental miRNAs could provide new avenues for the treatment of MIRI [9]. microRNA-146a (miR-146a) can regulate Nuclear Factor kappa-light-chain-enhancer of activated B cells (NF-κB) signaling pathway as a negative feedback regulator, which plays a significant role in the context of heart injury, especially through downregulating the expression of key adaptor proteins such as TNF receptor-associated factor 6 (TRAF6) and Interleukin-1 receptor-associated kinase 1 (IRAK1). Injection of miR-146a mimic during acute MI improved cardiac function, reduced scar mass, and increased viable heart tissue [10]. Intravenous administration of miR-146a mimic preserved cardiac function and reduced the infarction area through regulating the transcription of NOX4 [11]. Recently, a study reported that overexpression of miR-146a-5p suppressed hypoxia-induced cardiac fibrosis by inhibiting endothelial-to-mesenchymal transition in hypoxia human cardiac microvascular endothelial cells after MI [12]. However, the delivery of these molecules to the heart in a specific and efficient manner remains a challenge as extracellular RNAs are unstable and rapidly degraded.

Exosomes are small extracellular vesicles (EVs) that range in size from approximately 30 ~ 150 nanometers in diameter. Exosomes have been proposed as ideal delivery platform for miRNA-based therapy as miRNAs can be naturally transported within exosomes and exosomes can protect miRNAs from RNase degradation [13, 14]. Researchers have found that stem cell derived exosomes is a new hope for the treatment of cardiovascular disease [15, 16]. However, milk exosomes (MEs) are ideal drug delivery vehicles, even by oral administration, for miRNA-based therapy because the source is adequate and breast milk has unique health advantages for infants [17–19]. Interestingly, orally administered MEs carrying TNF-α siRNA could effectively reach colonic tissues and reduced TNF-α expression, thereby and alleviating colitis symptom in a murine model [20]. In addition, siRNA-Keap1 (siKeap1) was loaded into MEs and the obtained MEs-siKeap1 were found to significantly accelerate diabetic wound healing with enhanced neovascularization in a mouse model of diabetic wounds [21].

However, the intravenous delivery of exosomes into the lesion site remains a challenge due to poor targeting of unmodified exosomes. Strategies aimed at enhancing the targeting of drugs, such as miRNAs and siRNA, loaded in exosomes are increasingly paid attention. It was reported that hyaluronic acid (HA) modification could deliver drug-loaded MEs to the target cells, thus improving the anti-liver fibrosis effect of the drug [22]. Similarly, HA-modified MEs improved the biocompatibility stability and targeted transport properties of astaxanthin, thus inhibiting the expression of inflammatory factors and preventing the activation of macrophages [23]. In the context of tumor, MEs modified with integrin αVβ₃, αVβ5-binding peptide iRGD could successfully target lung adenocarcinoma cells [24]. The folate-modified MEs improved cellular uptake in cancer cells via folate receptor mediated endocytosis [25]. It has been demonstrated that the peptide sequence, CSTSMLKAC (IMTP), exhibited preferential binding to ischemic myocardium [26], and its effect has been verified after intravenous administration in the model of MIRI [27].

The aim of this study was to examine the effects of miR-146a containing MEs administrated by oral gavage on improvement of heart function after MIRI and whether miR-146a containing MEs could exert a better therapeutic role by modifying with IMTP on the surfaces after intravenous injection. In addition, the inhibition of NF-κB signaling pathway activation after the administration of miR-146a containing MEs was detected to further elucidate its mechanisms. Taken together, our findings suggested a cardioprotective effect of miR-146a containing MEs against MIRI, especially through enhanced targeting strategy, shedding light on an attractive therapeutic strategy for heart injury.

## Materials and methods

### Preparation of miR-146a-loaded MEs

All batches of MEs were isolated from 400 mL of commercial bovine milk. The milk was centrifuged at 10,000 ×g for 30 min at 4 °C to remove somatic cells and debris. The suspension was ultracentrifuged at 110,000 ×g (Hitachi, CS120FNX, Japan) for 70 min twice to remove fat globules, precipitated protein and larger vesicles. The exosome pellets were resuspended in phosphate-buffered saline (PBS), the isolated methods were modified from the literatures previous described [25, 28]. The total protein concentration was measured by BCA protein assay kit (Beyotime Biotechnology, Shanghai, China). Rno-miR-146a mimic (5’-UGAGAACUGAAUUCCAUGGGUU-3’) and negative control rno-miR-146a (miR1N0000001-1-5), were synthesized from RiboBio (Guangzhou, China). 1 µL rno-miR-146a mimic or negative control rno-miR-146a was resuspended in 199 µl PBS. Next, a volume of 200 was transferred between stainless steel plate electrodes separated with a 2 mm gap. A BTX Gemini X2 electroporator (Harvard Apparatus, USA) was used for pulsing, changing the voltage, and pulse duration. Electroporation conditions consisted of several parameters, and a square wave 1000 V voltage, 0.1 ms pulse duration was used for following experiments. After electrotransfection, the unencapsulated miR-146a was removed through a 10 kDa ultrafiltration tube (Millipore, Merck, Germany) via centrifugation at 3000 ×g for 10 min.

### Preparation of IMTP-miR-146a-loaded MEs

IMTP (Cat#EA-11-1, Ecbo Biotech, shanghai, China) and miR-146a-loaded MEs were conjugated via Michael addition reaction. Briefly, miR-146a-loaded MEs (750 µg), IMTP (100 µL) and reaction buffer (75 µL) were dissolved in 5 mL centrifuge tube. The mixture was incubated at 25 °C (250 rpm) for 3 h. The mixture was then incubated at 4 °C for 24 h. Then, using an ultrafiltration tube (100 kDa, UFC8100, Millipore) to remove unreacted substances and byproducts through centrifugation at 4000 ×g for 5 min. Finally, the IMTP-MEs-miR-146a was obtained and the optimal ratio of the reaction system was calculated.

### Characterization of MEs, MEs-miR-146a and IMTP-MEs-miR-146a

#### Transmission Electron Microscopy (TEM)

The Exosomes were resuspended in PBS before being assessed by TEM. A 10 µL sample was absorbed on a carbon-coated 200 mesh copper grid for 5 min, followed by another round of fixation with 2.5% (w/v) glutaraldehyde for 5 min. The grids were washed with PBS for 1 min. After negative staining with 2% (w/v) uranyl acetate for 1 min, the remaining dye was immediately removed with a filter paper. Thereafter, the grids were observed with TEM (Hitachi H-7650, Japan) operated at 120 kV.

#### Nanoparticle tracking analyses (NTA)

Quantification of the hydrodynamic diameter distribution and concentration (particle number/mL) of MEs was performed using Nanosight NS300 (Malvern Instruments, UK) equipped with a violet laser (405 nm) and running software version NTA3.4. The instrument was primed using PBS with pH 7.4, and the temperature was maintained at 25 °C. MEs were diluted in 0.22 μm PVDF membrane (13 mm, Millipore, Merck, Germany). Five measurements (60 s each) were obtained for each sample and the average was plotted as representation of size distribution and concentration (particles/mL).

#### Western blot analysis (WB)

Western blotting analyses were performed to detect typical exosome biomarkers (CD9, CD63, CD81 and TSG101) of MEs and NF-κB signaling pathway related proteins. Protein concentrations were determined using the BCA protein assay kit and 30 µg protein was loaded. All samples were prepared in 4× Laemmli sample buffer (Bio-Rad, USA) containing DTT and heated at 95 °C for 5 min. Samples were separated on 10% ExpressCast PAGE (New Cell & Molecular Biotech Co, Suzhou, China) and transferred to 0.45 μm PVDF membrane (Millipore, Germany). The membrane was then blocked with 5% Skim Milk (BD, USA) for 1.5 h at room temperature (RT) and incubated overnight at 4 °C with the primary antibodies. Secondary antibody reaction was conducted at RT for 1 h. Then, the immunoreactive protein bands were visualized using Enhanced Chemiluminescent (NCM, Suzhou, China) and images were taken by Tonon 5200 system (Shanghai, China). All the antibodies used in this study are listed in Supplementary Table 1.

### Cell culture

#### H9c2 cell culture

H9c2 cells were cultured in Dulbecco’s Modiffed of Eagle’s Medium (DMEM) with 1.5 g/L NaHCO3 (128-0001, Icell Bioscience Inc, Shanghai, China) supplemented with 10% fetal bovine serum (FBS, F0794, Gibico, Thermo Fisher Scientific, USA), and 1% penicillin streptomycin solution (30-002-CIa, Corning Cellgro) in a cell culture incubator with 37 °C and 5% CO_2_.

#### Primary neonatal cardiomyocyte culture

Neonatal rat cardiomyocytes (NRCMs) were isolated from P 0 rat as described previously [29] and cultured in DMEM with 4.5 g/L glucose (10-013-CV, Corning Cellgro, USA) with 10% FBS and 1% penicillin/streptomycin at 37 °C and 5% CO_2_.

### MEs labeling

For assessment of uptake and distribution of MEs, MEs were labeled using PKH26 Fluorescent Cell Linker Kit (UR52302, Umibio). 100 µL MEs and 50 µL PKH26 working solution were incubated at RT for 10 min. After that, 10 mL PBS was added for ultracentrifugation (UC), then the supernatant was discarded after centrifugation at 100,000×g for 70 min. Finally, the precipitate was resuspended with 200 µL PBS. H9c2 cells and NRCMs were already cultured with DMEM in 96 well plates (353,072, Corning Falcon) for 24 h. Immediately, the PKH26 labeled MEs (100 µg) transferred into 96 well plates in culture medium. Cells were cultured until 24 h prior to observe under the fluorescence microscope (IX51, Olympus, Japan).

### Oxygen glucose deprivation/reperfusion (OGD/R) model

The supernatant of H9c2 cells or NRCMs was refreshed with the glucose-free DMEM medium, and then the cells were placed in the anaerobic chamber, which introduced into a mixture containing 5% CO_2_ and 95% N_2_ for 4 h. The cells were divided into the Control group, OGD/R group, different concentration groups (0.1 µg/µL, 1 µg/µL, 5 µg/µL, 10 µg/µL, 20 µg/µL). After pretreatment for 24 h, the cells in each group except the Control group were subjected to OGD/R treatment.

### Cell counting kit-8 (CCK-8) assay

CCK-8 (40203ES80, Yeasen) was used to detect cell viability. The cells were seeded into 96-well plates at 8000 cells/well and cultured accordingly, followed by the addition of 10 µL CCK-8 solution. 2 h later, the OD value at 450 nm was read to measure the viability of the cells (Synergy 2, Bio Tek, USA).

### TdT-mediated dUTP nick end labeling (TUNEL)

The H9c2 cells and NRCMs in 24 well plates were fixed by 4% paraformaldehyde (PFA) for 30 min and subsequently permeabilized by 0.3% TritonX-100 for 5 min. The one-step TUNEL Apoptosis Detection Kit (C1088, Green Fluorescence, Beyotime) was used to incubate for 60 min at RT, following by nuclear labeling with DAPI (H-1200-10, Vector Laboratories, Burlingame, CA, USA), both in the dark. The staining was observed by fluorescence microscopy.

### Animals

Male SD rats (6–8 weeks old) were purchased from Shanghai SLAC Laboratory Animal CO.LTD (Shanghai, China), and they were bred at the Experimental Animal Center of Shanghai University of Traditional Chinese Medicine (SHUTCM). The rats were treated following the Guide for the Care and Use of Laboratory Animals as adopted and promulgated by the US NIH. They were placed in a specific-pathogen-free environment under a 12 h light/dark cycle, with access to feed and water. All animal experiments were approved by the Experimental Animal Center of SHUTCM and performed under the institutional guidelines for the care and use of laboratory animals (PZSHUTCM2212080003).

### Establishment of animal models

MIRI model was conducted according to our previous study [30]. Brifely, after anesthetizing with isoflurane (Bio-Rad), chest cavity was opened at the 4th intercostal space under mechanical ventilation. The left anterior descending (LAD) coronary artery was ligated for 30 min with a 6-0 silk suture. After closing thoracic incision, the animals were placed on a heated blanket until recovery from anaesthesia. Rats in MEs and MEs-miR-146a group were received oral administration of the drug (200 µg/200 g) once a day three days before I/R surgery. Rats in MEs-miR-146a and IMTP-MEs-miR-146a group were tail vein injection of the drug (100 µL/200 g) 5 min before the ischaemia was completed.

### Echocardiography

The rats were tested for cardiac function using a Vevo 2100 Imaging System (VisualSonics, USA) at 24 h after LAD ligation. Animals were anesthetized under isoflurane, and the heart was imaged in a 2-dimensional parasternal short-axis view. M-mode image of the left ventricle in the parasternal long-axis view was captured. Ejection fraction (EF) and shortening fraction (FS) were measured on the M-mode trajectory and data were collected for analysis.

### Hematoxylin-eosin (HE) staining

The isolated heart tissue was fixed in 4% PFA. After being dehydrated, paraffin embedded, the tissues were finally cut into 3 μm sections. HE staining of the sections was conducted in accordance with the standard experimental procedure.

### Quantitative real-time RT-PCR (qRT-PCR)

Heart tissue (50 mg) was extracted using NucleoZOL (MNG, Germany), and the purity and quality of total RNA were measured by Enzyme labeling instrument (Synergy 2, Bio Tek, USA). Complementary DNA was synthesized using miRcute Plus miRNA First-Strand cDNA Synthesis kit or FastKing gDNA Dispelling RT SuperMix (Tiangen, Beijing, China). qPCR array was determined using the miRcute Plus miRNA qPCR Detection Kit (Tiangen) following the amplification protocol: 95 °C, 15 min (1 cycle); 94 °C, 20 s, 60 °C, 34 s (40 cycles) or Hieff® qPCR SYBR Green Master Mix (No Rox) (Yeasen, Shanghai, China) following the amplification protocol: 95 °C, 5 min (1 cycle); 95 °C, 10 s, 60 °C, 30 s (40 cycles). Data analysis was based on the 2^−ΔΔCt^ method. The primers of genes were listed in Supplementary Table 2.

### Enzyme linked immunosorbent assay (ELISA) assay

The tumor necrosis factor-α (TNF-α) (ml002095), IL-1β (ml063132), IL-6 (ml063159), TGF-β (ml058986) and stromal cell derived factor-1 (SDF-1) rat ELISA kits were purchased from Shanghai Enzyme Linkage Biotech (Shanghai, China). IL-10 (BY-ER330194) and basic fibroblast growth factor (bFGF, BY-ER330506) ELISA kits were both purchased from Yabscience (Shanghai, China). The concentration of these cytokines was detected in the serum as per the manufacturer’s protocol.

### Oxidative stress indicator tests

The oxidative stress-related indicators in rat serum were determined through the Superoxide Dismutase (SOD) assay kit (WST-1 method) (A001-3-2) and the Lactate dehydrogenase (LDH) assay kit (A020-2-2), which were purchased from Nanjing Jiancheng bioengineering Institute (Nanjing, China).

### Creatine kinase MB isoenzyme (CK-MB) assay

The concentration of CK-MB in rat serum was measured CK-MB isoenzyme Assay Kit (H197-1-2, Nanjing Jiancheng bioengineering Institute) using 450 nm microplate spectrophotometers according to the standard instruction.

### Targeting of MEs to myocardial tissue

To track the targeting of MEs to myocardial tissue after modified with IMTP, the MEs were labeled with DiR (Umibio, UR21017). Then, the MIRI rats were randomized into five groups to receive PBS, DiR-labeled MEs, DiR-labeled MEs-miR-146a, DiR-labeled IMTP-MEs and DiR-labeled IMTP-MEs-miR-146a through the tail vein (200 µL). The distribution of MEs in major organs (heart, liver, spleen and kidney) was examined with an IVIS® Spectrum system (Caliper, USA) at 24 h after injection before echocardiography.

### Statistical analysis

Data were statistically analyzed using GraphPad Prism 8 and are expressed as the mean ± standard error (SEM). The significance was determined by one-way ANOVA and Tukey test. Statistical significance was set at *P* < 0.05.

## Results

### Preparation and characterization of MEs-miR-146a

**Fig. 1:**
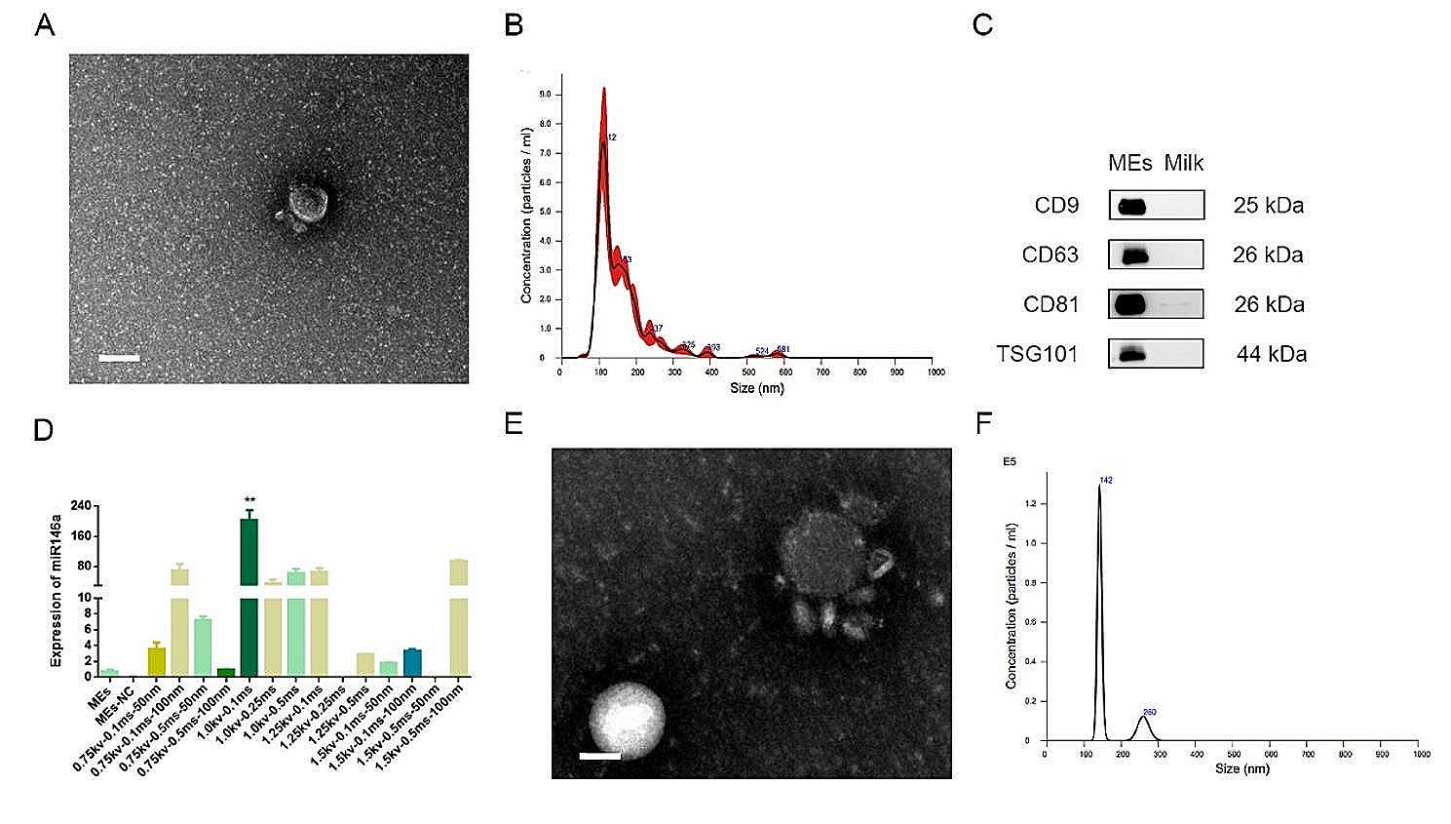
Preparation and characterization of MEs and MEs-miR-146a. (**A**) Representative TEM image of MEs. Scale bar = 100 nm. (**B**) Size diameters distribution of MEs as detected by NTA. (**C**) The typical exosome biomarkers of MEs and milk were measured by Western blotting. (**D**) The efficiency and the best parameters of MEs-miR-146a for electroporation were measured by qRT–PCR. (**E**) Representative TEM image of MEs-miR-146a. Scale bar = 100 nm. (**F**) Size of MEs-miR-146a detected by NTA

During the preparation of MEs-miR-146a, the MEs were characterized by TEM, NTA and Western blotting firstly. Under the TEM, MEs showed a typical disc-like bilayer structure (Fig. 1A). The results of NTA showed that the MEs have physically uniform particle size distributions, and the mean particle sizes of MEs was 112 nm (Fig. 1B). Western blotting analysis confirmed that MEs expressed the exosome markers CD9, CD63, CD81, TSG101, and milk only did not express the markers (Fig. 1C). Furthermore, to confirm that miR-146a was successfully loaded into the MEs, qPCR was used to detect the miR-146a content before and after electroporation, the data were shown in Fig. 1D. Comparing to MEs without miR-146a, electroconversion product showed a significant increase in miR-146 content at a square wave 1000 V voltage, 0.1 ms pulse duration, indicating that miR-146 mimics were successfully loaded into the MEs by electroporation. Subsequently, the structural and size of miR-146a-loaded MEs were determined. Both TEM and NTA results showed that the MEs maintained structural integrity before and after loading, and the size distribution of the diameters after loading was measured peaking at 142 nm (Fig. 1E, F). All the above results demonstrate that MEs-miR-146a was successfully prepared.

### MEs-miR-146a protected H9c2 and NRCMs from OGD/R induced damage

**Fig. 2:**
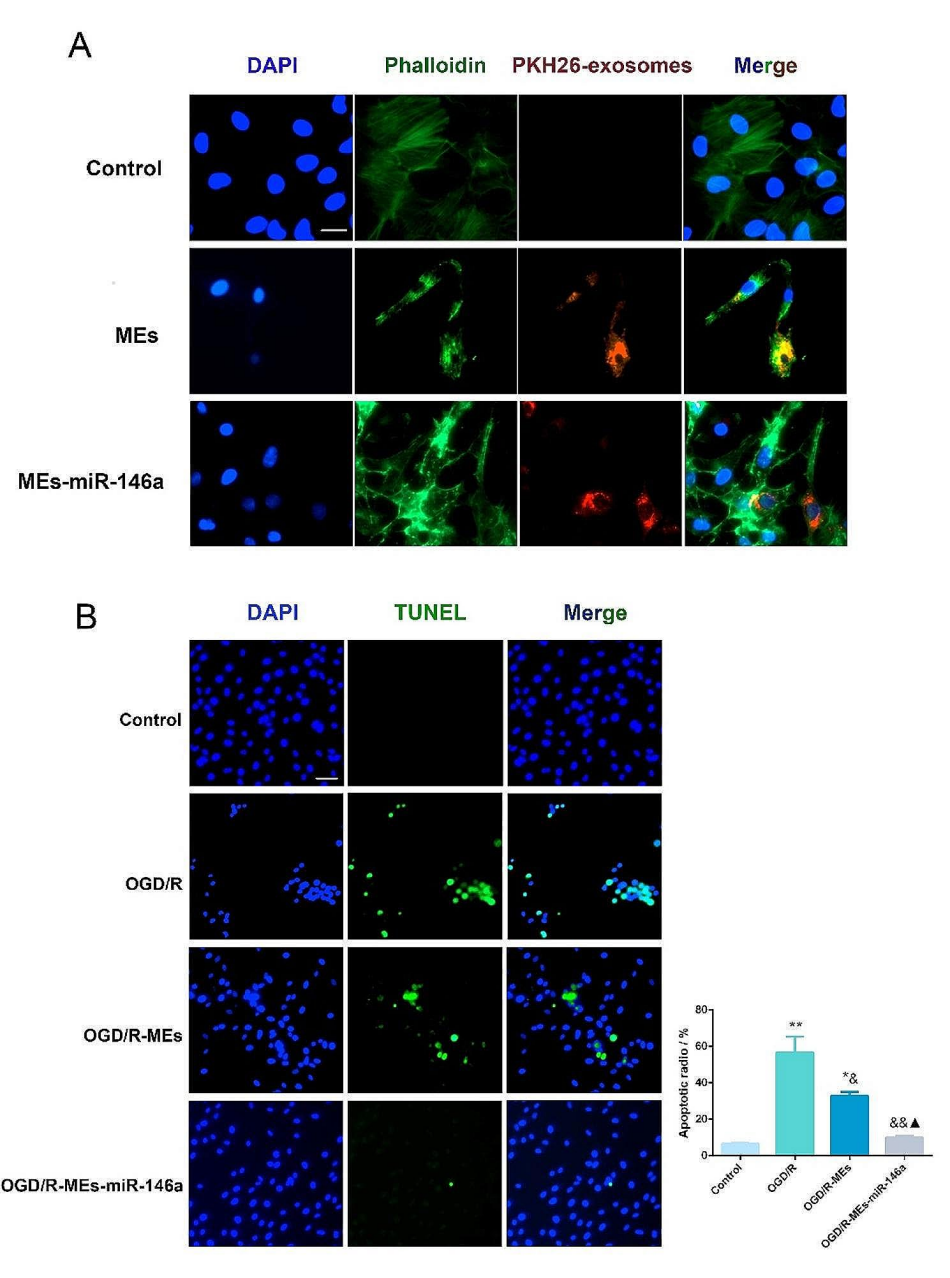
MEs-miR-146a protects H9c2 cells from OGD/R induced damage. (**A**) After 24 h of incubation, PKH26 labeled exosomes could be uptaken up by H9c2. Scale bar = 20 μm. (**B**) MEs-miR-146a decreased OGD/R induced cell apoptosis of H9c2 according to TUNEL assay. Scale bar = 50 μm. *P < 0.05, **P < 0.01 versus the control group; ^&^*P* < 0.05, ^&&^*P* < 0.01 versus the OGD/R group; ^▲^*P* < 0.05 versus the OGD/R-MEs group. *n* = 6

**Fig. 3:**
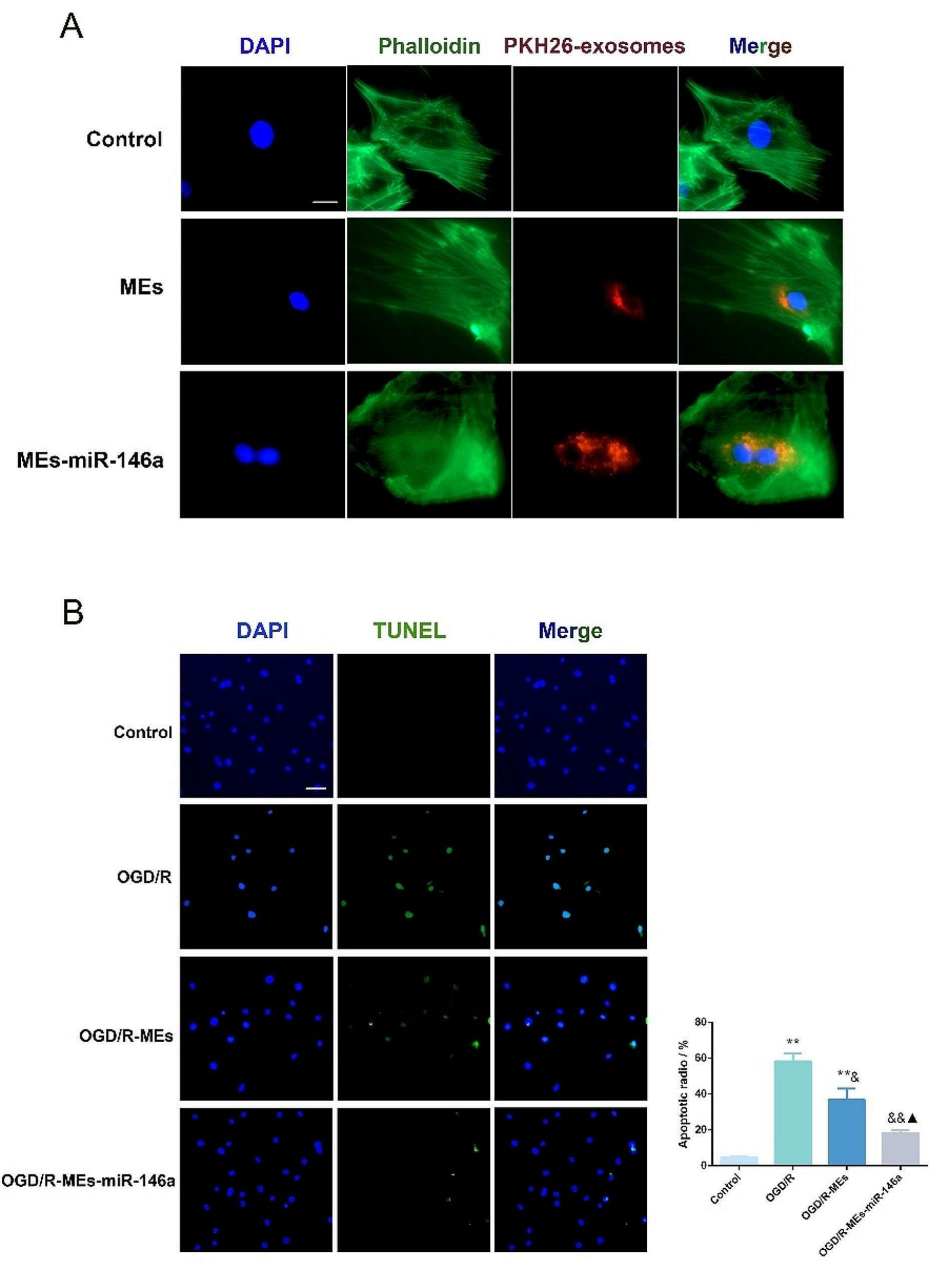
MEs-miR-146a protects NRCM cells from OGD/R induced damage. (**A**) Representative fluorescence images indicated that PKH26 labeled exosomes were taken up by NRCM cells after 24 h of incubation. Scale bar = 20 μm. (**B**) MEs-miR-146a decreased OGD/R induced cell apoptosis of NRCM according to TUNEL assay. Scale bar = 50 μm. **P < 0.01 versus the control group; ^&^*P* < 0.05, ^&&^*P* < 0.01 versus the OGD/R group; ^▲^*P* < 0.05 versus the OGD/R-MEs group. *n* = 6

Firstly, the cellular uptake of MEs-miR-146 in vitro was tested to evaluate the in vitro targeting ability of it. In brief, PKH26 labeled MEs-miR-146a were co-cultured with H9c2 or NRCMs, individually. After 24 h of incubation, PKH26-labeled exosomes could be taken up by H9c2 (Fig. 2A) and NRCM cells (Fig. 3A). The results suggested that MEs-miR-146a had a strong tendency to target myocardium in vitro. Secondly, an in vitro cellular injury model was established both in H9c2 and NRCMs in a mixture containing 5% CO_2_ and 95% N_2_ for 4 h. We found that both MEs and MEs-miR-146a at a concentration of 1 µg/µL had the best protective effect according to the results of the CCK-8 assay (Fig. S1). We further examined the cardioprotective effects of MEs and MEs-miR-146a on H9c2 (Fig. 2B) and NRCM (Fig. 3B) by TUNEL staining, OGD/R treatment caused plenty of apoptotic H9c2 and NRCM cells. However, cell apoptosis was obviously reduced in MEs-miR-146a treatment group.

### Oral administration of MEs-miR-146a improved cardiac function of rat after MIRI

**Fig. 4:**
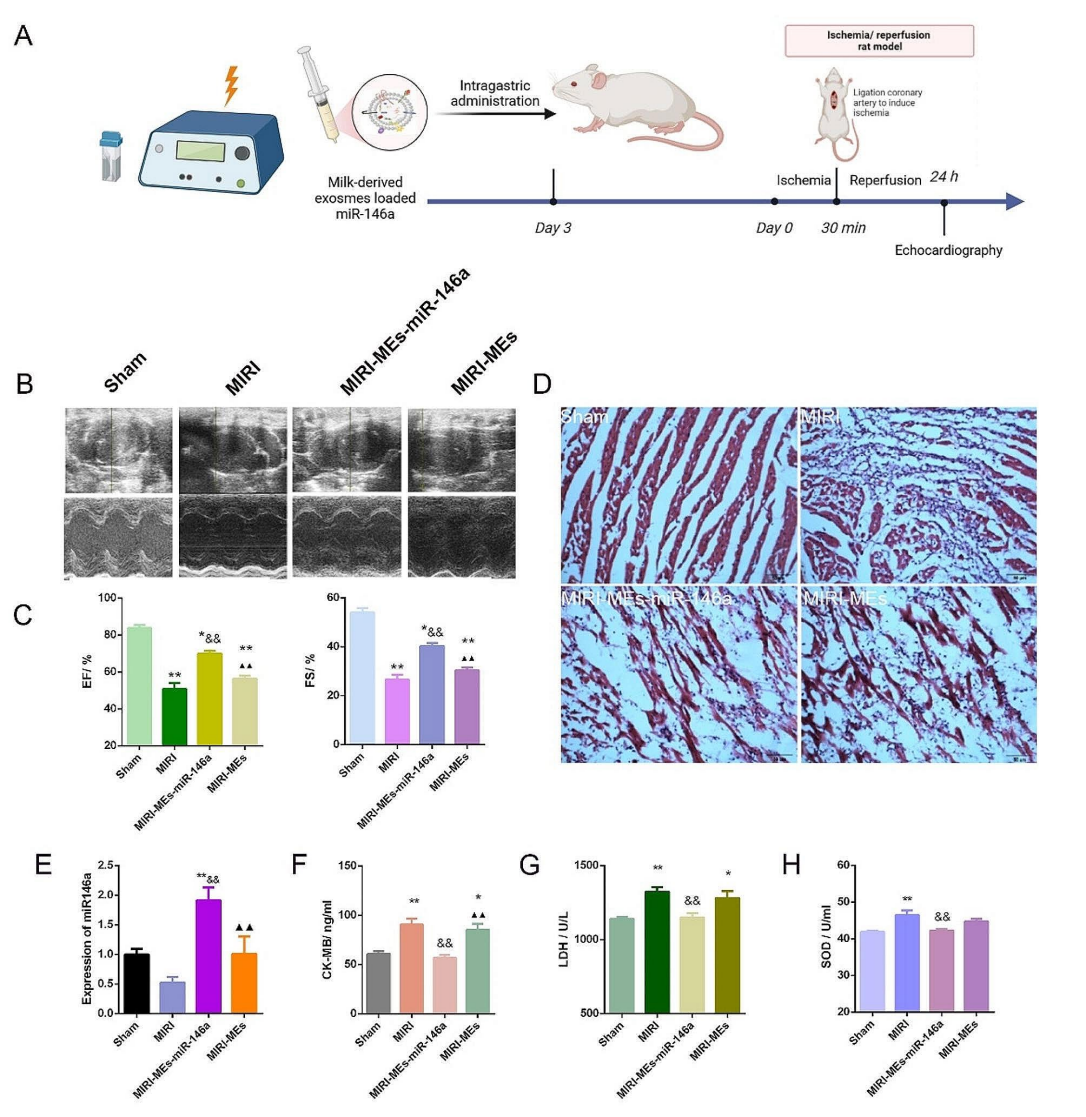
Oral administration of MEs-miR-146a improved cardiac function after MIRI. (**A**) The preparation flow of MEs-miR-146a and the schematic illustration of the treatment plan. (**B**) Representative echocardiography images. (**C**) The values of EF and FS. *P < 0.05, **P < 0.01 versus the Sham group; ^&&^*P* < 0.01 versus the MIRI group; ^▲▲^*P* < 0.01 versus the MIRI-MEs-miR-146a group. (**D**) Representative H&E images of heart tissue from each group. Scale bar = 50 μm. (**E**) Detection of miR-146a expression in rat myocardium by qRT-PCR. (F-H) Quantification of CK-MB, LDH and SOD levels in the rat serum after MIRI. *P < 0.05, **P < 0.01 versus the Sham group; ^&&^*P* < 0.01 versus the MIRI group; ^▲▲^*P* < 0.01 versus the MIRI-MEs-miR-146a group. *n* = 5

To evaluate the in vivo cardioprotective effects of MEs-miR-146a, a rat model of MIRI was established as illustrated in Fig. 4A. Because MEs have the advantage of passing through the gastrointestinal tract without being decomposed by gastric acid, we chose oral administration three days ago before establishing MIRI model. To observe whether the MEs-miR-146a can effectively protective myocardial injury after oral administration, the small animal echocardiography was used 24 h after reperfusion to evaluate the heart function. MIRI rats were divided into Sham, MIRI, MIRI-MEs-miR-146a and MIRI-MEs groups. Compared to the MIRI and MIRI-MEs groups, the EF and FS values of MIRI rats from the MEs-miR-146a group were increased (Fig. 4B, C). Meanwhile, the left ventricular end-diastolic dimension (LVEDd), left ventricular end-systolic dimension (LVEDs), left ventricular end-diastolic volume (LVEDV) and left ventricular end-diastolic volume (LVESV) values were improved (Fig. S2). What’s more, HE staining suggested that MEs-miR-146a group can reduce inflammatory cell infiltration (Fig. 4D). Next, we examined the level of miR-146a in heart tissue to confirm whether MEs-miR-146a could effectively deliver miR-146a. The results showed that the content of miR-146a was significantly increased (Fig. 4E). What’s more, the CK-MB, SOD, LDH concentration, which are indicators of myocardial injure, were reduced in MEs-miR-146a group (Fig. 4F-H).

### Oral administration of MEs-miR-146a decreased myocardial tissue apoptosis and expression of inflammatory factors

**Fig. 5:**
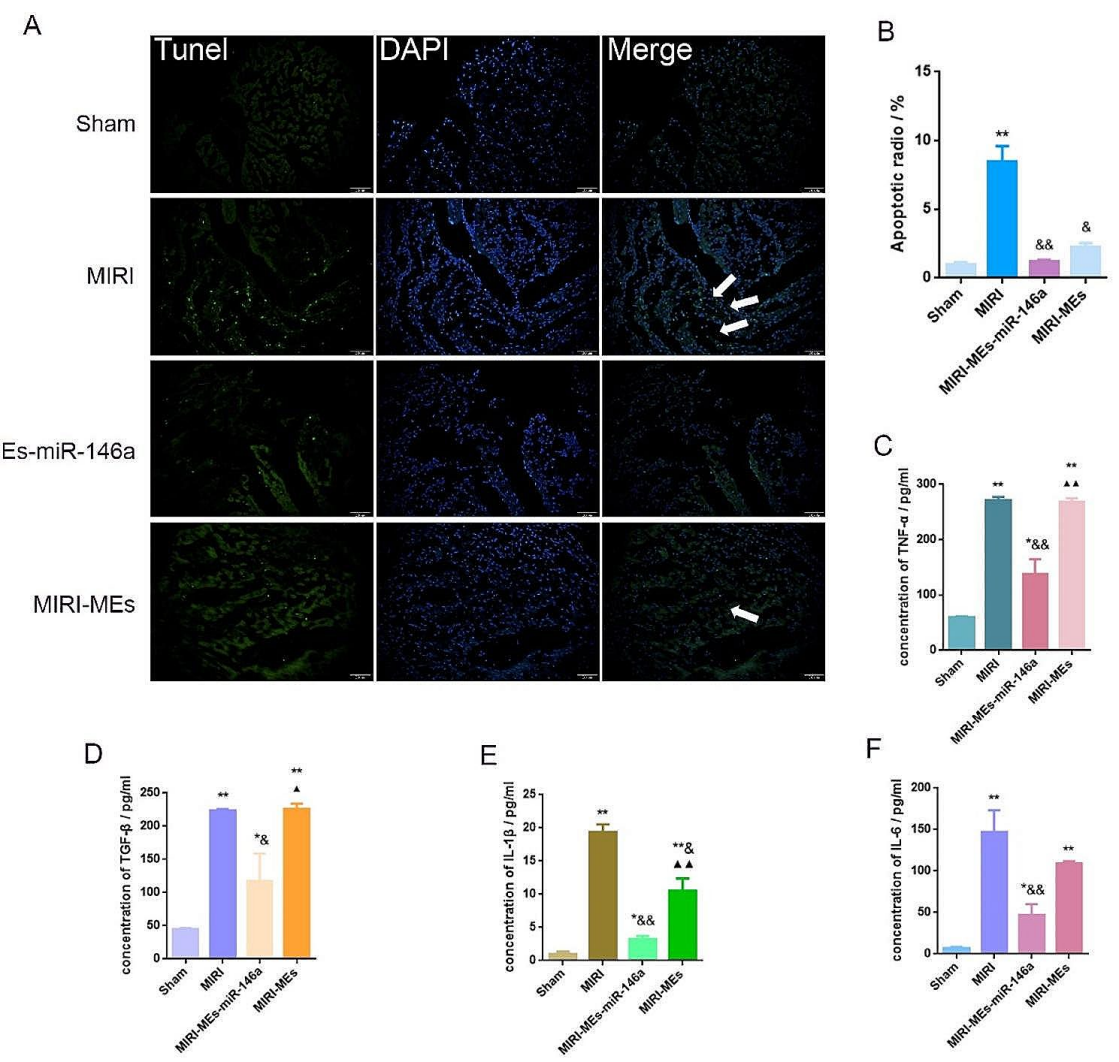
MEs-miR-146a exhibited better therapeutic efficacy in decreasing apoptotic cells and limiting inflammation than MEs. (**A**-**B**) Representative TUNEL staining images and quantification of apoptotic radio. **P < 0.01 versus the Sham group; ^&^*P* < 0.05 and ^&&^*P* < 0.01 versus the MIRI group. (**C**-**F**) The expression of inflammatory factors of rat serum were detected by ELISA. *P < 0.05, **P < 0.01 versus the Sham group; ^&^*P* < 0.05, ^&&^*P* < 0.01 versus the MIRI group; ^▲^*P* < 0.05, ^▲▲^*P* < 0.01 versus the MIRI-MEs-miR-146a group. *n* = 5

TUNEL staining was performed to determine the number of apoptotic cells in the infarct area. The ratio of apoptotic cells was significantly lower in the MIRI-MEs-miR-146a group than in the MIRI and MIRI-MEs groups (Fig. 5A, B). We also detected the expression of apoptosis-related proteins, including Cleaved caspase-3, Bax and Bcl-2. The administration of MEs-miR-146a increased the expression of Bcl-2 and Bcl-2/Bax compared with the other two groups (MIRI and MIRI-MEs groups) in the injured myocardium (Fig. S3). In addition, TNF-α expression in injured myocardium of MIRI-MEs-miR-146a group were lower than those in the other three groups, as well as TGF-β, IL-1β and IL-6 levels (Fig. 5C–F), which suggested that MEs-miR-146a treatment could attenuate the inflammatory response after MIRI.

### MEs-miR-146a protected myocardium from MIRI through suppressing NF-κB activation and IRAK1 / TRAF6 expression

**Fig. 6:**
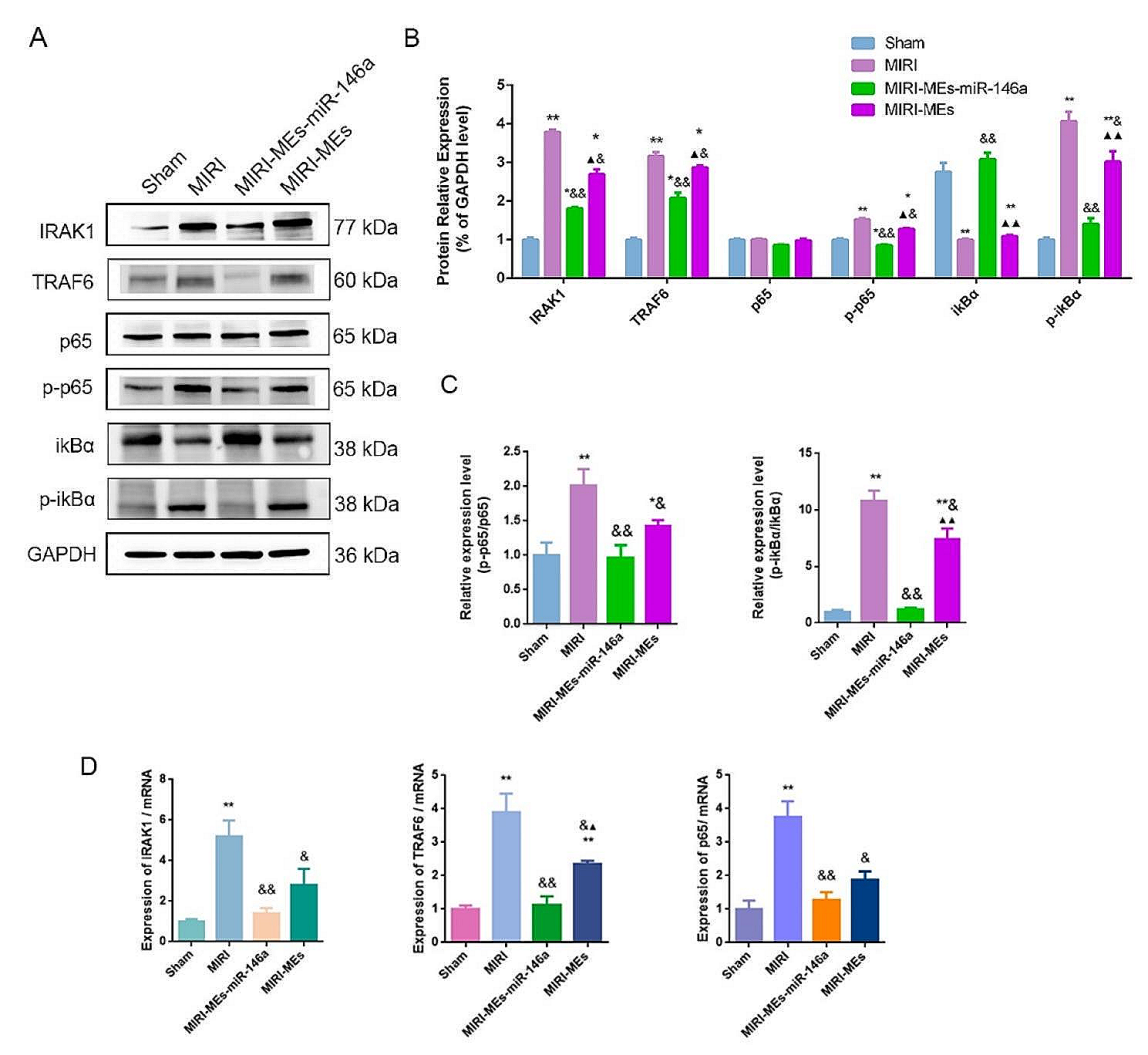
MEs-miR-146a protected myocardium from MIRI through suppressing NF-κB activation and IRAK1 / TRAF6 expression. (**A**-**C**) Changes in intracellular IRAK1, TRAF6, p65, p-p65, ikBα and p-ikBα protein levels after treatment of MEs-miR-146a. (**D**) The expression of IRAK1, TRAF6 and p65 mRNA level were examined by qRT-PCR. *P < 0.05, **P < 0.01 versus the Sham group; ^&^*P* < 0.05, ^&&^*P* < 0.01 versus the MIRI group; ^▲^*P* < 0.05, ^▲▲^*P* < 0.01 versus the MIRI-MEs-miR-146a group. *n* = 5

We then explored whether MEs-miR-146 treatment could regulate NF-κB signaling pathway through targeting of IRAK and TRAF6. Comparing to the MIRI and MIRI-MEs groups, MEs-miR-146a treatment reduced p-p65 and p-ikBα protein levels in the injured myocardium after administration. Interestingly, IRAK1 and TRAF6 protein levels were alleviated to some extent after MEs-miR-146a treatment (Fig. 6A-C). The IRAK1, TRAF6 and p65 mRNA level were detected by q-PCR. The data showing that after MEs-miR-146a treatment, all the three levels were decreased more than those in MIRI-MEs group, especially the level of TRAF6 was lower compared with MIRI-MEs group with statistically significant difference (Fig. 6D).

### Successful construction of IMTP-MEs-miR-146a

**Fig. 7:**
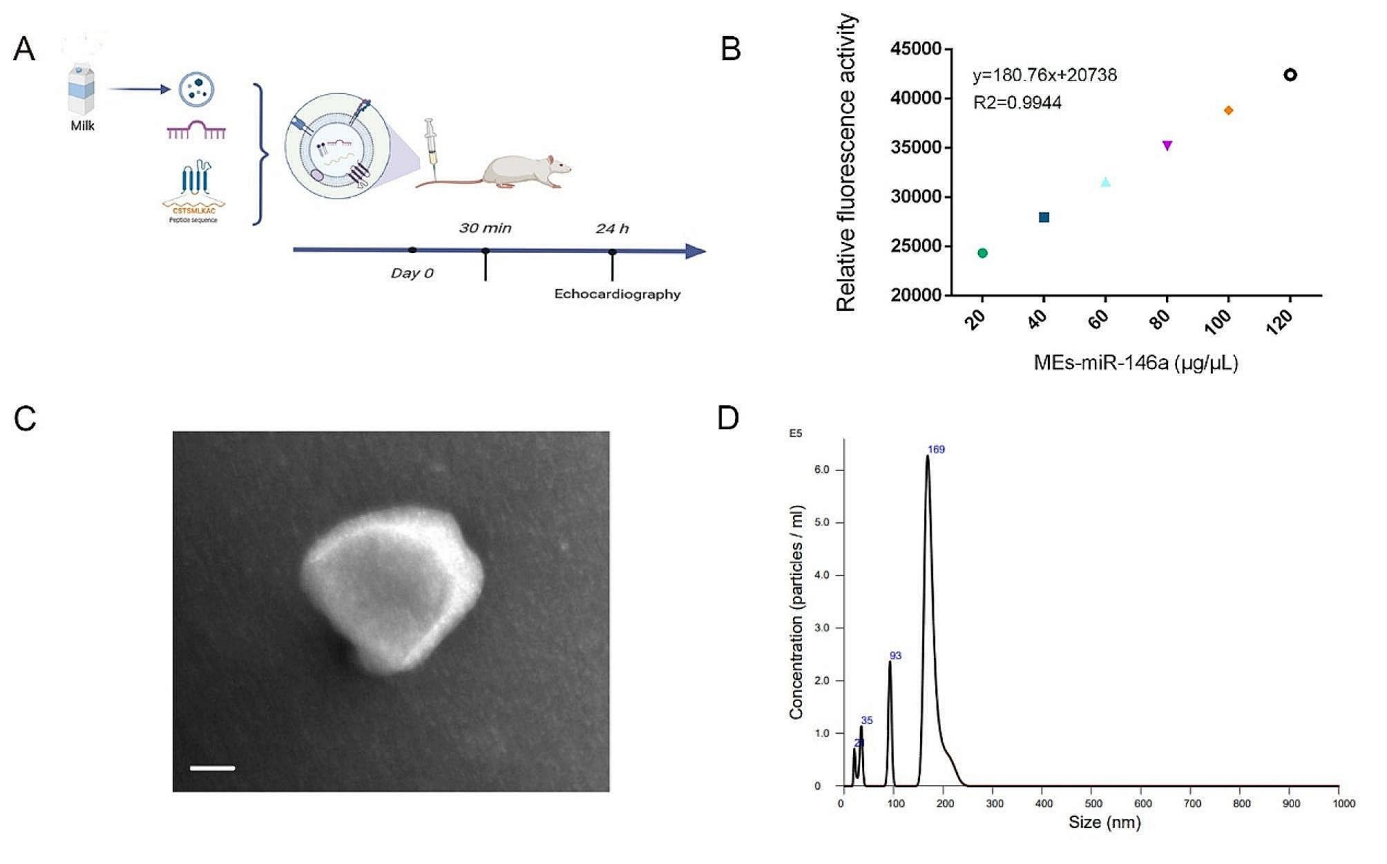
Preparation and characterization of IMTP-MEs-miR-146a. (**A**) Schematic of the IMTP-MEs-miR-146a used for MIRI therapy. (**B**) A standard curve was generated based on different IMTP-MEs-miR-146a concentrations and their corresponding fluorescence intensities. (**C**) The morphology of IMTP-MEs-miR-146a was observed by TEM. Scale bar = 100 nm. (**D**) Characterization of IMTP-MEs-miR-146a by NTA

The target IMTP-MEs-miR-146a were prepared as illustrated in Fig. 7A. Briefly, miR-146a mimic was encapsulated into MEs by an electroporation approach as we previously described. Then, IMTP was synthesized through Michael addition reactions. To identify the optimal formulation to modify MEs-miR-146a with IMTP, different ratios of MEs-miR-146a and IMTP were mixed and reacted. The relative fluorescence intensity of each group was measured by a multifunctional microplate reader after ultraffitration purification. A standard curve was generated based on different MEs-miR-146a concentrations and their corresponding fluorescence intensities (Fig. 7B). TEM showed that the diameter of IMTP-MEs-miR-146a was slightly larger than that of unmodified MEs, but it still maintained a typical exosomal structure (Fig. 7C). Meanwhile, the average size of IMTP-MEs-miR-146a was approximately 169 nm (Fig. 7D), which is slightly larger than that of MEs and MEs-miR-146a (112 nm and 142 nm) (Fig. 1B, F).

### Enhanced accumulation of IMTP-MEs in myocardial tissue

**Fig. 8:**
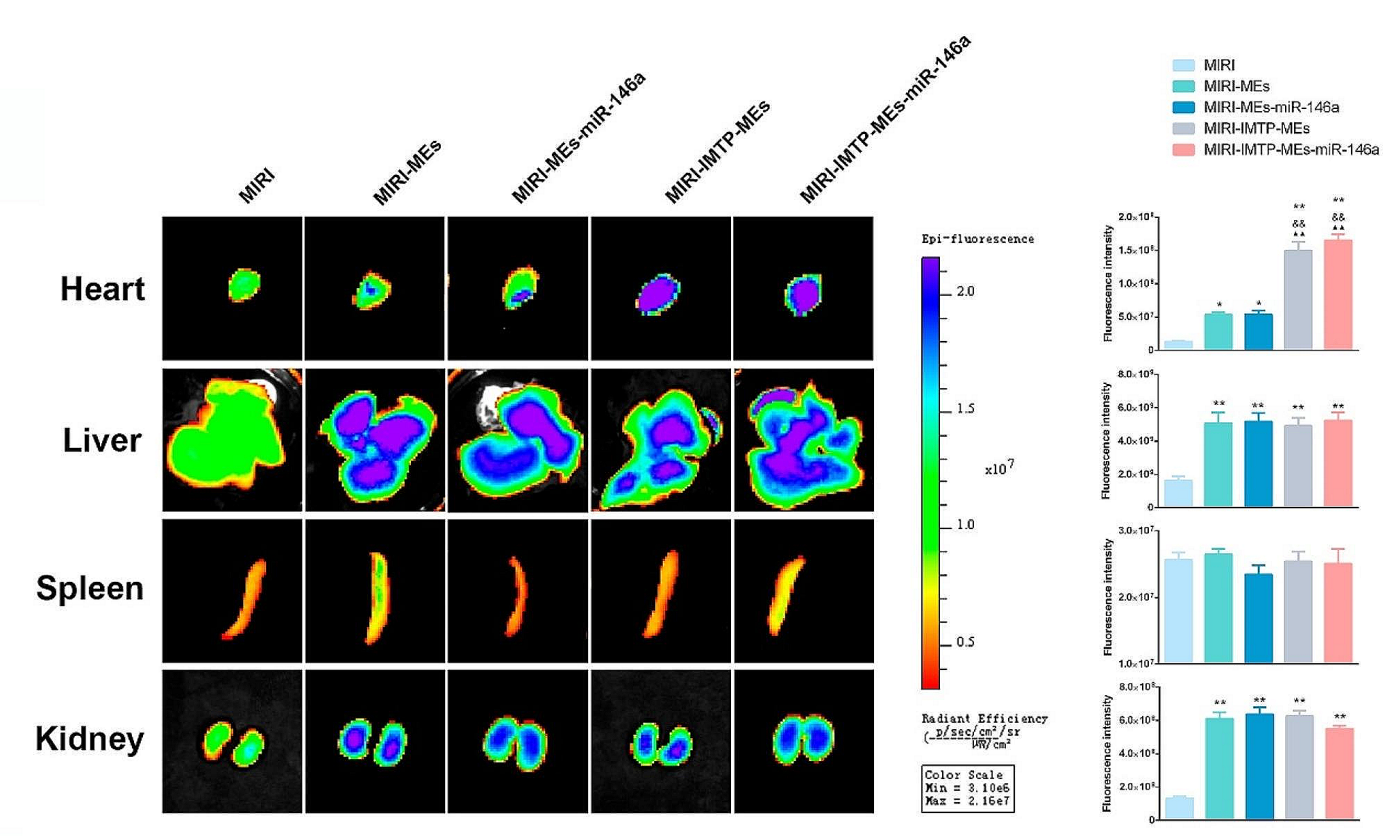
The biodistribution of MEs in MIRI rat. In vitro imaging analyses of organs 24 h after the intravenous injection of MEs. *P < 0.05, **P < 0.01 versus the MIRI group; ^&&^*P* < 0.01 versus the MIRI-MEs group; ^▲▲^*P* < 0.01 versus the MIRI-MEs-miR-146a group

To determine the in vivo biodistribution of IMTP-MEs, DiR-labeled modified MEs were intravenously injected into SD rat. Notably increased accumulation of IMTP-MEs and IMTP-MEs-miR-146a were observed in heart compared with that of undecorated MEs (Fig. 8). Not only, we also evaluated the biodistribution of MEs in other different organs (liver, spleen and kidney), and we found that the difference was not statistically significant. Together, these results suggest that IMTP modified MEs promote the accumulation of MEs in myocardial tissue. Besides, we examined the cardiac function in each group by echocardiography, and the data confirmed that IMTP-MEs-miR-146a played the best therapeutic effect (Fig. S4).

### Intravenous injection of IMTP-MEs-miR-146a improved cardiac function in rats after MIRI

**Fig. 9:**
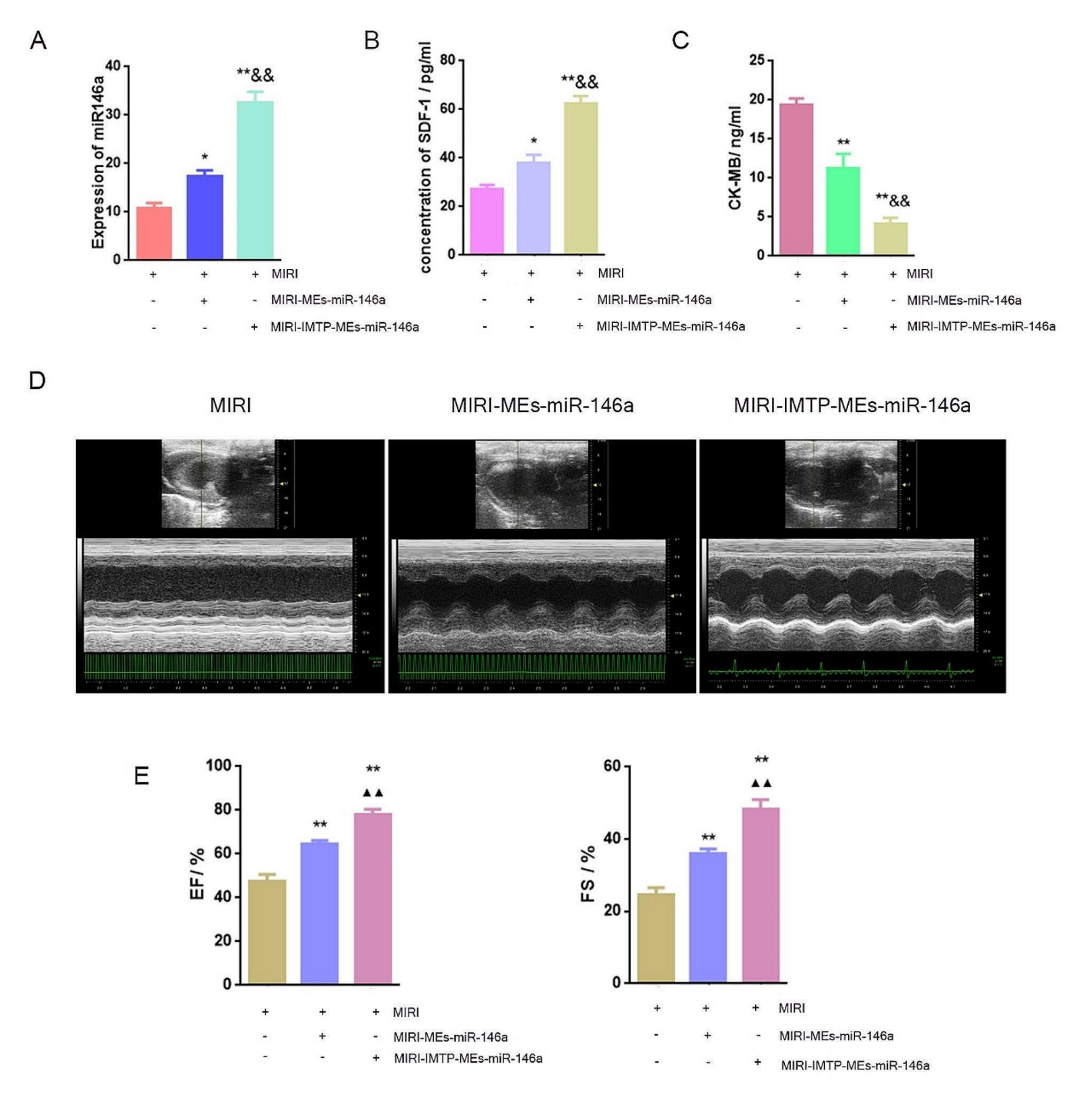
Tail vein injection of IMTP-MEs-miR-146a exhibited superior therapeutic efficacy in improving cardiac function. (**A**) Quantification of miR-146a mRNA level in myocardial tissue. *P < 0.05, **P < 0.01 versus the MIRI group; ^&&^*P* < 0.01 versus the MIRI-MEs-miR-146a group. *n* = 8. (**B**-**C**) The expression of SDF-1 and CK-MB in the rat serum were detected by ELISA. *P < 0.05, **P < 0.01 versus the MIRI group; ^&&^*P* < 0.01 versus the MIRI-MEs-miR-146a group. *n* = 8. (**D**-**E**) Representative echocardiography images from each group and quantitative analysis of EF and FS. **P < 0.01 versus the MIRI group; ^▲▲^*P* < 0.01 versus the MIRI-MEs-miR-146a group. *n* = 8

Next, we focused on the differences between MEs-miR-146a treatment and IMTP-MEs-miR-146a treatment after intravenous injection. To examine whether modified MEs-miR-146a could increase the level of miR-146a in injured myocardium after intravenous injection, we detected miR-146a expression by q-PCR firstly. We found that the miR-146a level of modified MEs-miR-146a group was significantly increased than the MEs-miR-146a group, which indicated that IMTP modification enhanced the targeting delivery of miR-146a to the heart (Fig. 9A). Then, SDF-1 activity was examined in rat serum using ELISA. Significant increase of SDF-1 expression was found in IMTP-MEs-miR-146a group, and which was higher than that in the unmodified MEs-miR-146a group (Fig. 9B). Additionally, we investigated the level of myocardial injury biomarker, CK-MB, in rat serum. Both MEs-miR-146a and IMTP-MEs-miR-146a reduced the CK-MB level, this decrease was more pronounced after the treatment of MEs-miR-146a modified with IMTP (Fig. 9C). Echocardiography was utilized to assess heart function and as shown in Fig. 9D-E and Figure S5, MIRI was linked to significant decreases in EF and FS, and although MEs-miR-146a treatment elevated the value of EF and FS, IMTP-MEs-miR-146a could further increase the value of EF and FS.

### Intravenous injection of IMTP-MEs-miR-146a reduced myocardial tissue apoptosis and suppressed inflammation

**Fig. 10:**
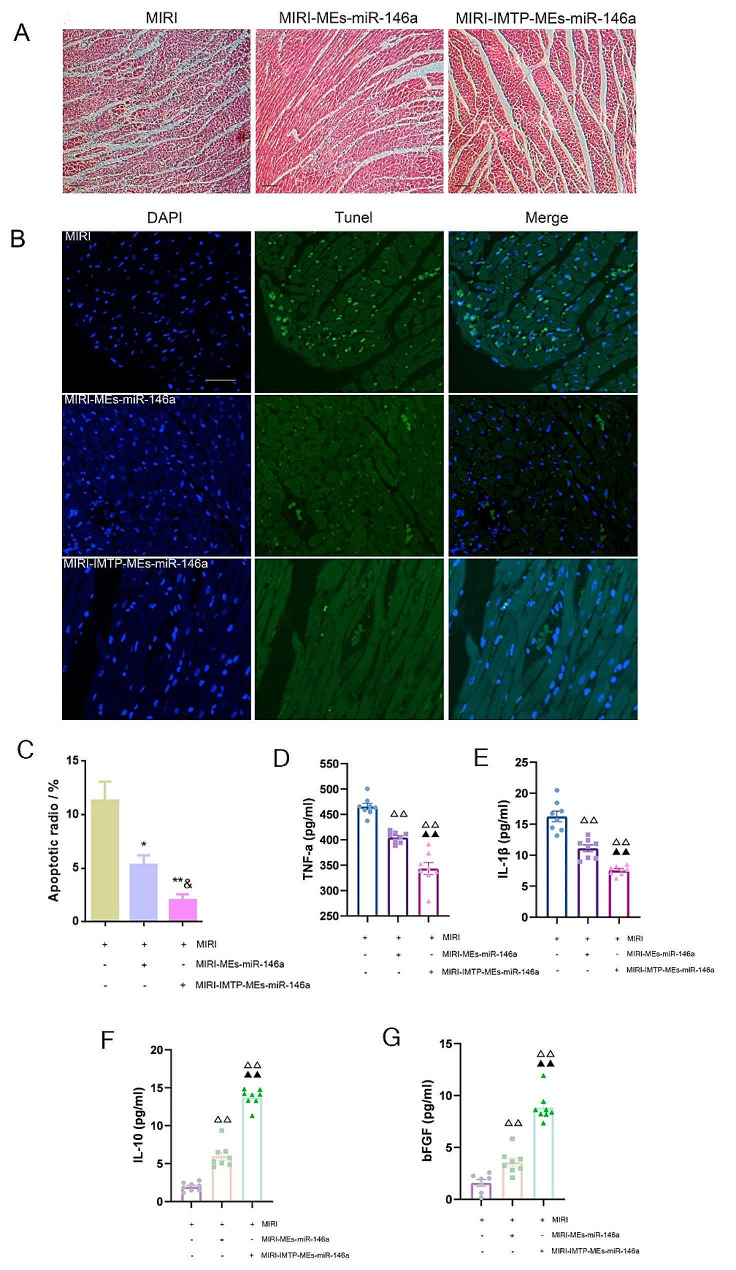
Tail vein injection of IMTP-MEs-miR-146a significantly ameliorated myocardium apoptosis and attenuated inflammation at the early stage of MIRI. (**A**) Representative H&E images of heart tissue from each group. Scale bar = 50 μm. (**B**-**C**) Representative images of TUNEL staining 24 h after MIRI and quantitative analysis of apoptotic radio. *P < 0.05 and **P < 0.01 versus the MIRI group; ^&^*P* < 0.05 versus the MIRI-MEs-miR-146a group. *n* = 8. (**D**-**G**) The expression of inflammatory factors of rat serum were detected by ELISA. ^∆∆^*P* < 0.01 versus the MIRI group; ^▲▲^*P* < 0.01 versus the MIRI-MEs-miR-146a group. *n* = 8

Previous studies have established that myocardial inflammation and cardiomyocyte death are primary pathological changes in MIRI. HE staining showing that IMTP-MEs-miR-146a treatment more effectively relieved the inflammatory cell infiltration than the MEs-miR-146a (Fig. 10A). Furthermore, TUNEL assay indicated that MIRI resulted in substantial cardiomyocyte apoptosis, whereas this apoptotic rate was reduced approximately 10% after administration of MEs-miR-146a modified with IMTP, and approximately 6% in the group of MEs-miR-146a, compared with the MIRI group (Fig. 10B, C), which revealed that IMTP-MEs-miR-146a significantly reduced cell apoptosis after MIRI. The protein expression of Cleaved caspase-3, Bax and Bcl-2 was further detected. Injection of IMTP-MEs-miR-146a obviously decrease the Cleaved caspase-3 and Bax protein levels compared with the MIRI-MEs-miR-146a group, and increased the Bcl-2 protein level more markedly as shown in Figure S6. In addition, the pro-inflammatory levels of TNFα and IL-1β were much lower after IMTP-MEs-miR-146a treatment than those in MEs-miR-146a group (Fig. 10D, E). Interestingly, the anti-inflammatory factor such as IL-10 expression in rat serum of modified MEs-miR-146a was higher than that in the MIRI and MIRI-MEs-miR-146a (Fig. 7F). Moreover, compared to the MIRI and MIRI-MEs-miR-146a groups, IMTP-MEs-miR-146a injection up-regulated the level of bFGF, which may be beneficial for the survival of cardiomyocytes (Fig. 10G). These data thus supported anti-inflammatory and anti-apoptotic actions of IMTP-MEs-miR-146a during MIRI.

### Intravenous injection of IMTP-MEs-miR-146a protected myocardium from MIRI through targeting IRAK1/TRAF6 and suppressing NF-κB activation

**Fig. 11:**
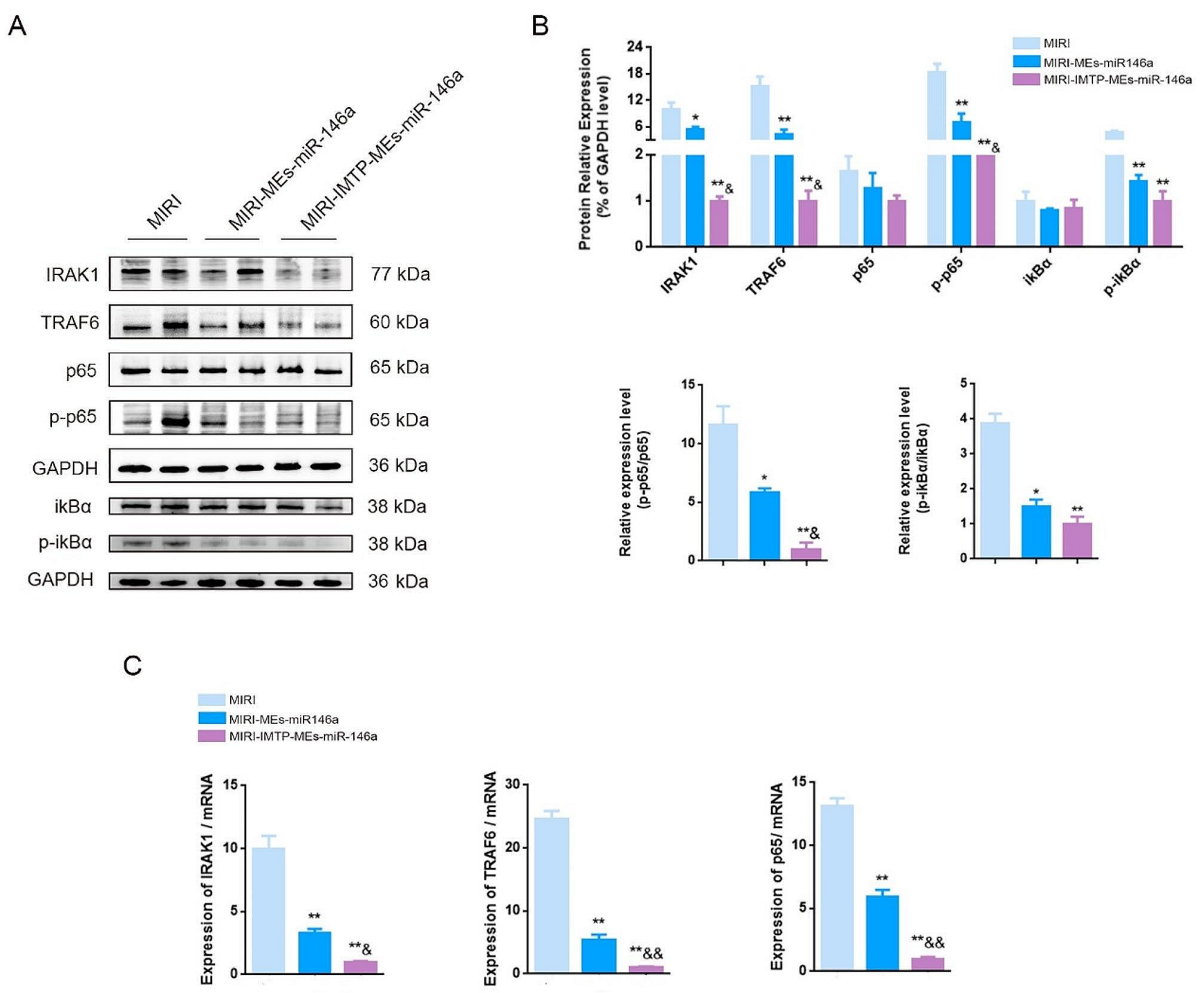
Intravenous injection of IMTP-MEs-miR-146a protected myocardium from MIRI through targeting IRAK1/TRAF6 and suppressing NF-κB activation. (**A**-**B**) Changes in intracellular IRAK1, TRAF6, p65, p-p65, ikBα and p-ikBα protein levels after treatment of MEs-miR-146a and IMTP-MEs-miR-146a. (**C**) The expression of IRAK1, TRAF6 and p65 mRNA level were detected by qRT-PCR. *P < 0.05, **P < 0.01 versus the MIRI group; ^&^*P* < 0.05, ^&&^*P* < 0.01 versus the MIRI-MEs-miR-146a group. *n* = 8

We previously demonstrated that miR-146a in myocardial tissue can regulate NF-κB signaling pathway through targeting of IRAK and TRAF6. Compared with MEs-miR-146a, the levels of IRAK1, TRAF6, p-p65 and p-ikBα protein in the group of modified MEs-miR-146a were lower. MEs-miR-146a treatment reduced the level of IRAK and TRAF6, while the decrease was more significant in IMTP-MEs-miR-146a group (Fig. 11A, B). Although intravenous injection of MEs-miR-146a obviously down-regulated the expression of IRAK1 and TRAF6 mRNA, IMTP-MEs-miR-146a treatment further reduced their levels (Fig. 11C). Therefore, we proposed that IMTP-MEs-miR-146a exerted their anti-inflammatory effect and reduced cell apoptosis by inhibiting the IRAK1/TRAF6/NF-κB signaling pathway.

## Discussion

The prevention and treatment of MIRI is an area of active research. After MIRI, several outcomes can occur, including myocardial stunning, microvascular obstruction, arrhythmias, cardiac dysfunction, even death. Treating MIRI involves strategies to minimize the damage caused by the restoration of blood flow after a period of ischemia. Some of the current pharmacological agents include antioxidants, calcium channel blockers, nitric oxide donors and anti-inflammatory drugs. In this study, we delivered miR-146a to injured myocardium through MEs and found that miR-146a containing MEs could attenuate the heart injury and improve the cardiac function. Most importantly, the improvement was much more obvious when the miR-146a containing MEs were modified with myocardial targeted peptide IMTP on their surfaces. Therefore, we suggested a potential strategy for the treatment of MIRI. This therapy is worth promoting because the MEs, as a drug deliver platform, could be easily harvested and has low toxicity and immunogenicity [31]. However, further human experiments are still needed to verify their immune response in the human body.

miRNAs are small, non-coding RNAs that regulate gene expression post-transcriptionally. miR-146a is known to play a significant role in regulating the immune response and inflammation, and has been studied for its role in various physiological and pathological processes, including inflammation, cancer, and cardiovascular diseases. In the context of MI, miR-146a is of particular interest due to its involvement in the modulation of inflammatory responses and its potential impact on the progression of heart disease. Expression of miR-146a was significantly upregulated in ST-segment elevation myocardial infarction (STEMI) patients, thus it can serve as a biomarker for adverse prognosis of STEMI [32, 33]. However, the expressions of miR-146a-5p was 4.048-fold lower in patients with STEMI compared to the control group patients according to another study carried out by Bukauskas et al. [34]. Exosomes derived from miR-146a-modified adipose-derived stem cells decreased myocardial damage via downregulation of EGR1 after MI [35]. The combination of miR-21 and miR-146a could effectively protect against cardiac ischemia/hypoxia-induced apoptosis, which was mediated by the p-p38-caspase-3 signaling pathway [36]. In the context of MIRI, it has been found that miR-146a significantly decreased MIRI-induced myocardial infarct size and prevented MIRI-induced decreases in heart function [37]. In addition, miR-146a mimic preserved cardiac function and decreased the inflammatory response by inhibiting NOX4/P38 signaling pathway in mice with MIRI [11]. We found that miR-146a delivered by MEs could reduce myocardial injury and improve heart function after MIRI, which was consistent with reports from other laboratories. It’s worth noting that while the regulatory role of miR-146a in inflammation and its potential therapeutic implications for myocardial injury are promising, further research is needed to fully understand its mechanisms and to translate these findings into clinical applications.

Recently, exosome-based therapy has emerged as one of the most potential strategies to promote heart repair after myocardial injury. Exosomes have the natural ability to deliver molecules between cells, which is being harnessed to develop exosome-based drug delivery systems. Recent evidences indicated that miRNA play an important role in exosome-mediated cardioprotection after MI. Mesenchymal stem cell (MSC)-derived exosomes carrying specific miRNAs could promote cardiac regeneration and repair through anti-apoptotic, anti-fibrotic, anti-inflammatory, and angiogenic effects [38–41]. It has been found that MSC-derived exosomes carrying miR-182-5p improved heart function and reduced inflammation and cell pyroptosis by targeting GSDMD in mice [42]. Besides, exosomes containing miR-126 and miR-146a mimics reduced infarct size and improved angiogenesis and was desirable for heart regeneration after MI [43]. MEs, specifically, are derived from the mammary gland cells and are present in the milk of various species, including humans and cows. The interest in MEs has increased in recent years due to their roles in immune system modulation, and MEs may be involved in the delivery of bioactive compounds to the infant during breastfeeding. Additionally, MEs are good candidates for drug delivery systems, due to their natural origin and the ability to encapsulate and protect drugs or other therapeutic molecules. The suitability of MEs as delivery vehicles for miRNAs has been proved to be feasible [44]. The accumulation of DiR-labeled MEs could be found in the heart after intravenous and oral administration [45]. In addition, miRNAs delivered by MEs accumulated in intestinal mucosa, spleen, liver, heart or brain after oral gavage of MEs [45]. Research indicated that bovine MEs alleviated cardiac fibrosis and enhanced the heart function via enhancing angiogenesis in cardiac fibrosis rat [46]. Human breast milk-derived exosomal miR-148a-3p exerted an important role on reducing necrotizing enterocolitis by regulating p53 and Sirtuin 1 [47]. In addition, Yan et al. [48] proved that the miR-31-5p loaded in MEs was able to resist degradation, thus promoting the angiogenesis and enhancing diabetic wound healing. In our study, we found that MEs loaded with miR-146a through electroporation decreased apoptosis of neonatal rat cardiomyocytes and H9c2 under OGD treatment in vitro. Furthermore, MEs loaded with miR-146a decreased heart injury and improved cardiac function after oral administration. Therefore, oral gavage of MEs with cardioprotective drugs may be a potential strategy for the treatment of MIRI and is worth further research. Based on this, continued advancements in understanding the biology of MEs, along with developments in nanotechnology and drug delivery systems, will be key to unlocking their potential in treating MIRI. While the idea of using MEs for myocardial injury is promising, it is important to note that research in this area is still in the early stages.

Despite the promising potential of MEs in therapeutics and regenerative medicine, several challenges remain, including standardization of exosome isolation and characterization methods, and target to the injured tissues after systemic administration. Up to date, several strategies have been investigated to enhance the target of exosomes to the injured tissues. One of the strategies is to restructure transmembrane proteins of exosomes to fuse with homing peptides or ligands, which is conducive to the targeting capability of exosome to tissues or organs carrying the corresponding receptors [49, 50]. To enhance the target of EVs into the ischemic brain, targeting ligands were attached onto EVs. These modified EVs could target the lesion region of the ischemic brain after intravenous administration and signifcantly suppressed the inflammatory response [51]. Hyaluronic acid-coated bovine MEs was able to specifically target CD44-positive cancer cells and significantly increased antitumor efficacy without systemic toxicity [52]. In previous study, we increased the targeting of MSC-exosomes to brain through modifying the surfaces of exosomes with RVG peptide, which can interact specifically with the acetylcholine receptor to enable viral entry into brain. We found that intravenous injection of RVG-modified MSC-exosomes improved cognitive function and reduced the expression of pro-inflammatory cytokines in APP/PS1 mice [53]. Researchers have discovered that the peptide sequence, CSTSMLKAC (IMTP), can preferentially target to ischemic myocardium region [26]. In terms of the MIRI, IMTP has been applied to targeted therapy, which resulting in high localization within the ischemic zone after systemic administration [27]. MSC-derived exosomes have been reconstructed with IMTP and their therapeutic efficacy was elucidated in MI. MSC‐derived IMTP‐exosomes remarkably suppressed inflammation and cardiomyocyte apoptosis and improved cardiac function [54]. In our study, we applied the IMTP onto milk exosomal surface to target ischemic myocardium. Our results demonstrated that IMTP‐MEs loaded with miR-146a could remarkably suppress inflammation and cardiomyocyte apoptosis, reduce heart injury, and improve heart function in a rat MIRI model.

Although IMTP modified and miR-146a loaded MEs showed great therapeutic potential for MIRI, the mechanism of action still needs further clarification. miR-146a targets and downregulates several key molecules in the NF-κB signaling pathway, which is a critical regulator of the inflammatory response. By modulating this pathway, miR-146a can potentially reduce excessive inflammatory responses following myocardial injury, which may help in reducing tissue damage and promoting healing. During myocardial I/R injury, the role of NF-κB becomes particularly critical due to its involvement in the inflammatory response and regulation of apoptotic pathways. By inducing the expression of pro-inflammatory cytokines and chemokines, NF-κB contributes to the recruitment of inflammatory cells to the ischemic-reperfused myocardium, exacerbating tissue damage. Gao et al. [55] proposed that miR-146a attenuated sepsis-induced cardiac dysfunction by preventing NF-κB activation and inflammatory cytokine production via targeting of IRAK and TRAF6. It has been reported that miRNA-146a protected myocardium from MIRI through suppressing NF-κB activation and IRAK1 and TRAF6 expression [37]. Dexmedetomidine decreased myocardial infarction size and cell apoptosis through miR-146a-3p targeting IRAK1 and TRAF6 via inhibition of the NF-κB pathway [56]. Interestingly, Tongxinluo-pretreated MSCs markedly promoted cardiac repair through the exosomal transfer of miR-146a-5p targeting IRAK1/NF-κB p65 pathway [57]. We found that miR-146 treatment through MEs or IMTP modified MEs could regulating NF-κB signaling pathway through targeting of IRAK and TRAF6. As much more miR-146a was delivered into the injury site after intravenous injection of IMTP modified MEs, the suppression of NF-κB pathway was more obvious, which then decreased inflammatory reaction and improved cardiac function. However, more studies are needed to fully understand the roles of miR-146a carried in IMTP modified MEs in cardiovascular diseases such as MIRI.

However, there are some limitations in this study. The therapeutic effects and optimal dosages of IMTP modified and miR-146a loaded MEs should be investigated in large mammal animal models. Whether through oral application or tail vein injection, as both are performed before reperfusion. It is necessary to use the same treatment after MIRI in future studies to verify its effectiveness, in order to provide more data for clinical application. In addition, the preclinical and in vitro studies are needed to understand how these exosomes can be effectively targeted to the heart. Lastly, further research is needed to fully understand the safety profiles and the long-term outcomes of modulating miR-146a levels through IMTP modified MEs in patients with MIRI.

## Conclusions

In this study, we showed the therapeutic effects of miR-146a containing MEs on MIRI. The miR-146a containing MEs administrated by oral gavage decreased the level of inflammatory factors and showed beneficial effects in improvement of heart function after MIRI. IMTP functionalized MEs enhanced the targeting delivery of miR-146a loaded in MEs to the site of myocardial injury and showed effective suppression of inflammatory response, thus exerting better therapeutic role after systemic administration. IMTP-decorated MEs can be proven to be a practical drug delivery system of miRNA for targeted heart injury therapy. Moreover, miR-146a containing MEs could negatively regulate NF-κB signaling pathway and reduced the expression of TRAF6 and IRAK1. While there were several concerns that still required further investigation, our study proposed a promising therapeutic strategy for MIRI treatment using miR-146a and targeting peptide functionalized MEs. To the best of our knowledge, this is the first time MEs were reconstructed with IMTP to achieve targeted delivery of miRNA molecules to ischemic myocardium after MIRI.

### Electronic supplementary material

Below is the link to the electronic supplementary material.

Supplementary Material 1

## Data Availability

The data that support the findings of this study are available from the corresponding author upon reasonable request.
