# Supplementary material for: Targeting delivery of miR-146a via IMTP modified milk exosomes exerted cardioprotective effects by inhibiting NF-κB signaling pathway after myocardial ischemia-reperfusion injury
Source: J Nanobiotechnology. 2024 Jul 1;22:382. doi: 10.1186/s12951-024-02631-0 (PMC11218161; doi:10.1186/s12951-024-02631-0)
Supplement: Supplementary file 1 — Supplementary Material 1 [file 12951_2024_2631_MOESM1_ESM.docx]

**Supplementary Materials**


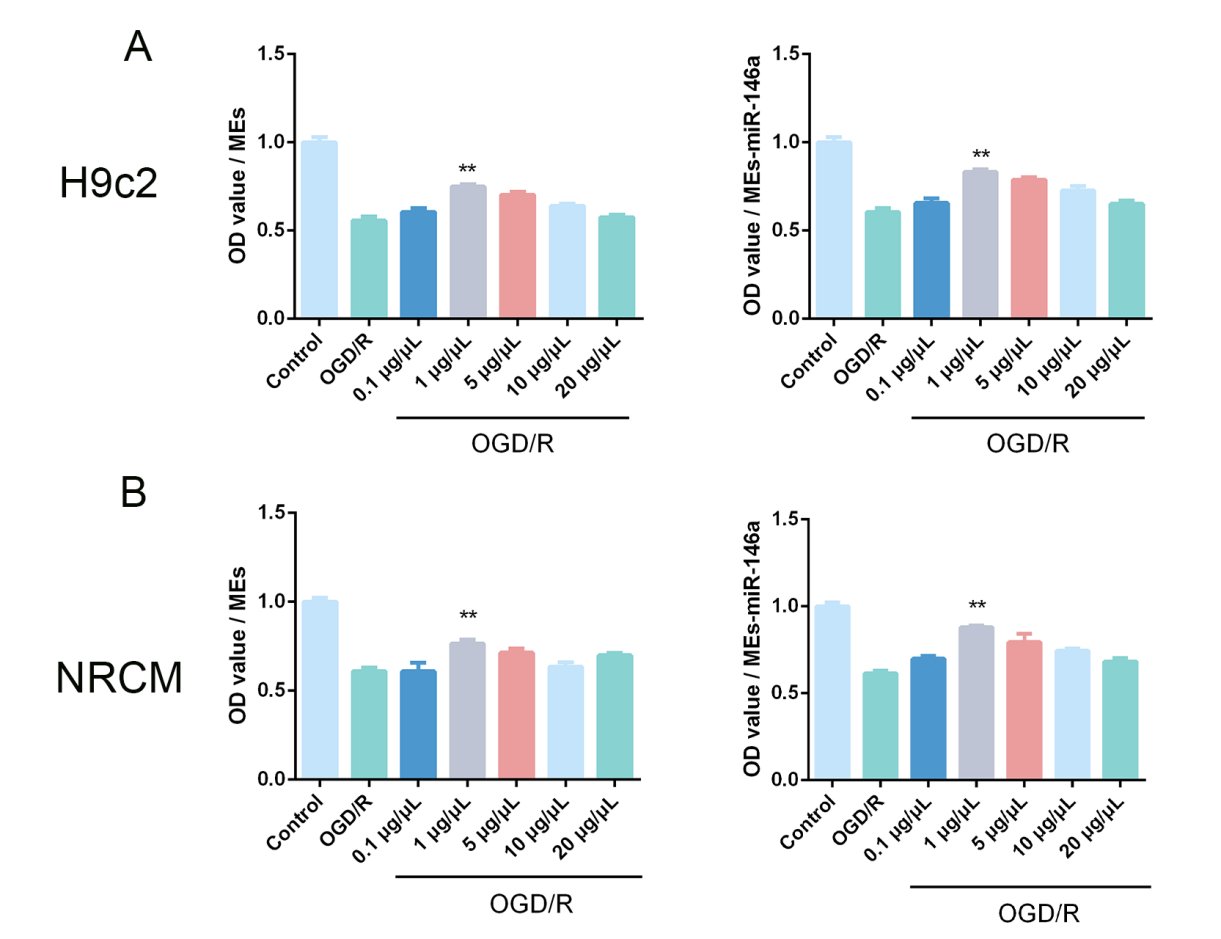


Figure S1. 1 μg/μL MEs and MEs-miR-146a protected H9c2 and NRCM cells from OGD/R induced damage. (A) Protective effects of MEs and MEs-miR-146a at different concentrations on H9c2 after OGD/R induced damage by CCK-8 assay. (B) Protective effects of MEs and MEs-miR-146a at different concentrations on NRCM cells after OGD/R induced damage by CCK-8 assay. ***P* < 0.01 versus the OGD/R group. n = 6.

**
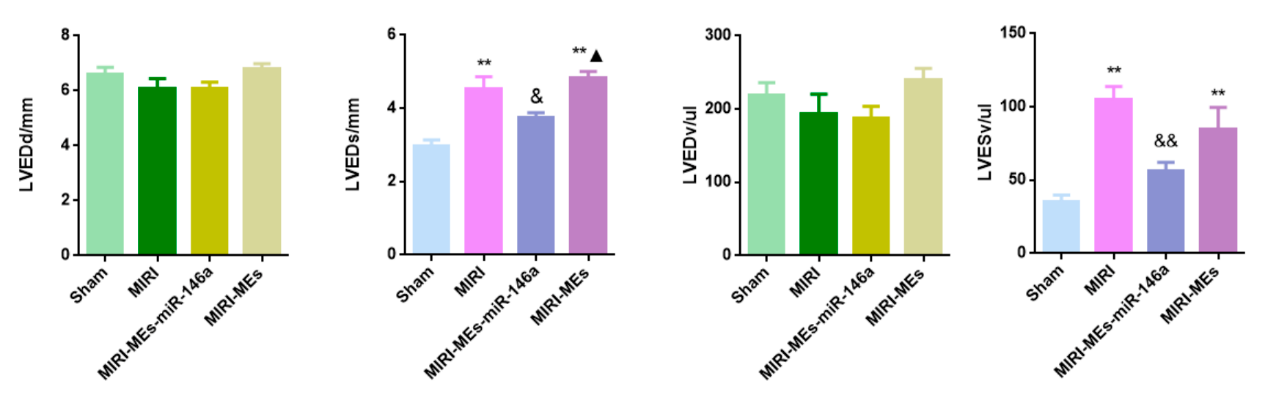
**

Figure S2. Quantitative analysis of LVEDd, LVEDs, LVEDv, LVESv at 24 h after MIRI according to echocardiography. ***P* < 0.01 versus the Sham group; ^&^*P* < 0.05, ^&&^*P* < 0.01 versus the MIRI group; ^▲^*P* < 0.05 versus the MIRI-MEs-miR-146a group. n=5.

**
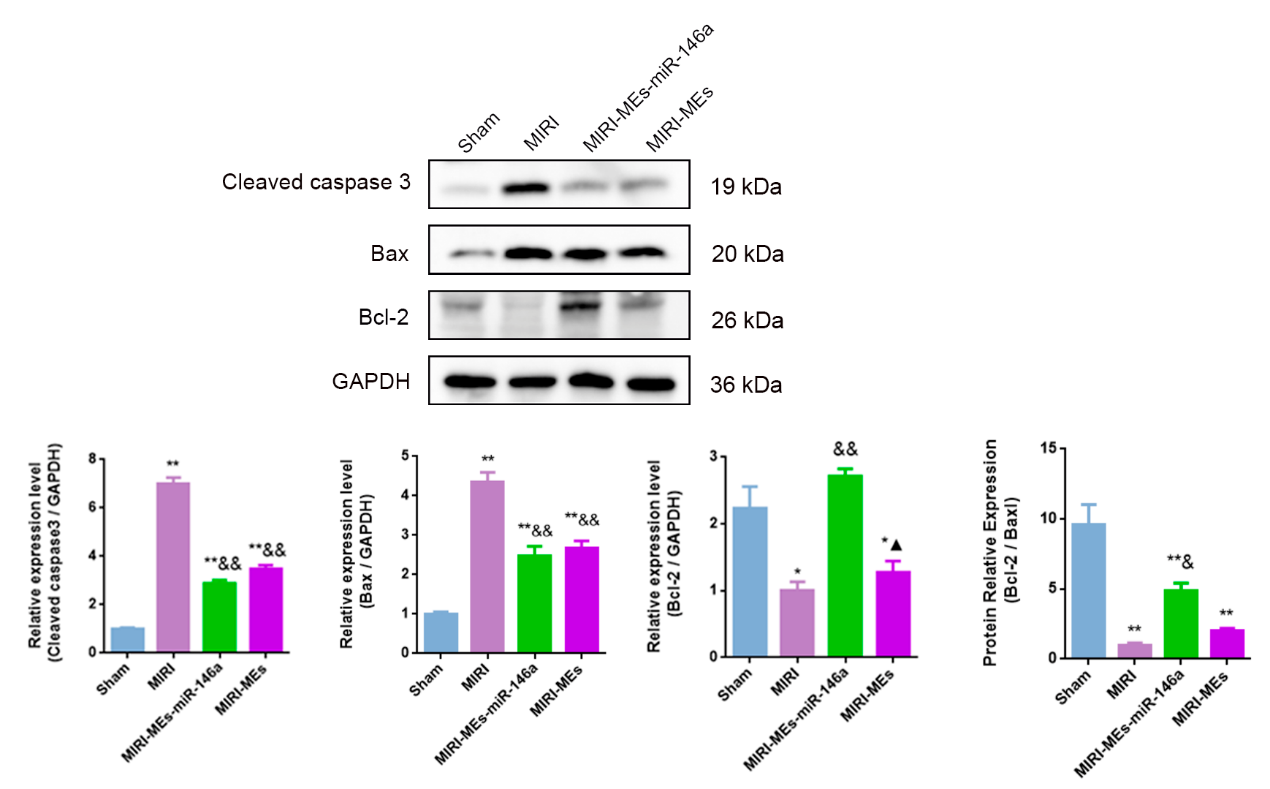
**

Figure S3. Representative western blotting bands and quantification of Cleaved-caspase 3, Bax and Bcl-2 level after oral administration of MEs-miR-146a. **P* < 0.05, ***P* < 0.01 versus the Sham group; ^&^*P* < 0.05, ^&&^*P* < 0.01 versus the MIRI group; ^▲^*P* < 0.05 versus the MIRI-MEs-miR-146a group. n=3.

**
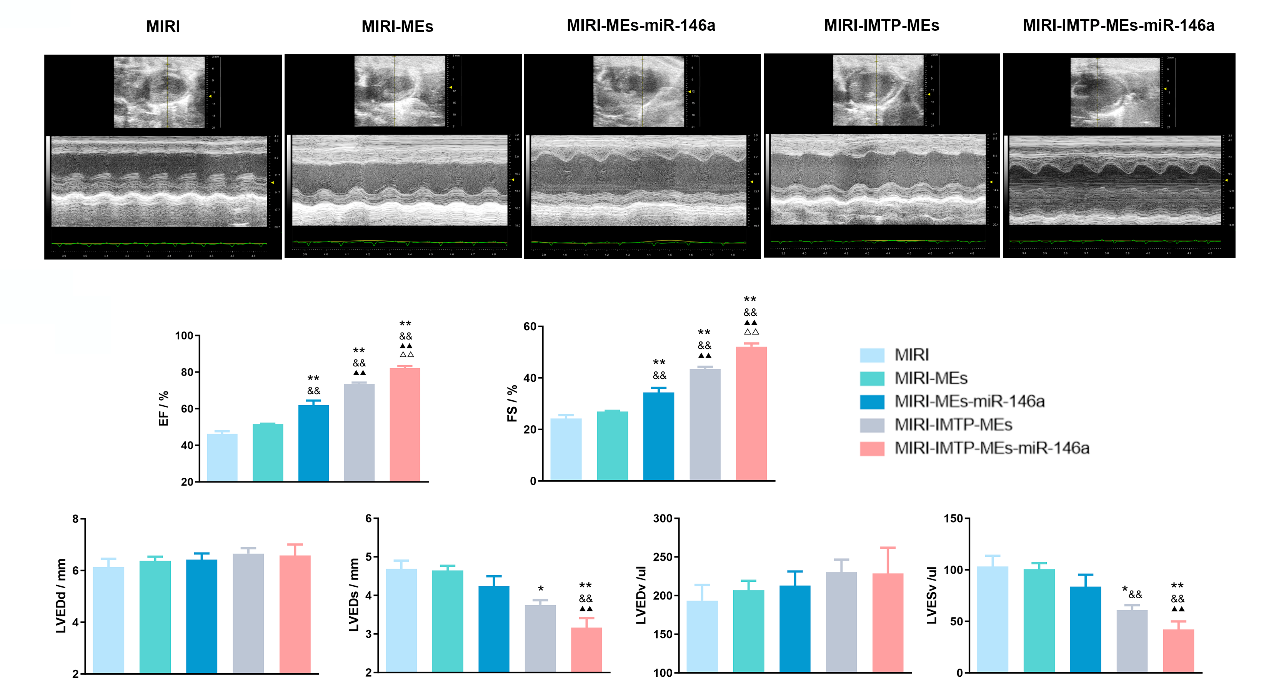
**

Figure S4. Rat echocardiography results. (A) Representative echocardiography images of each group. (B) Quantitative analysis of EF, FS, LVEDd, LVEDs, LVEDv, LVESv at 24 h after MIRI. **P* < 0.05, ***P* < 0.01 versus the MIRI group; ^&&^*P* < 0.01 versus the MIRI-MEs group; ^▲▲^*P* < 0.01 versus the MIRI-MEs-miR-146a group; ^△△^*P* < 0.01 versus the MIRI-IMTP-MEs group.

**
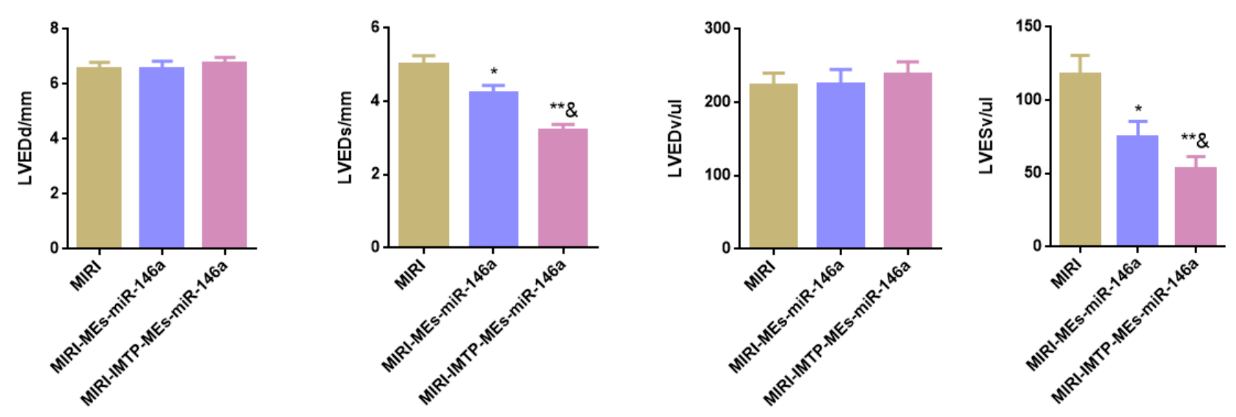
**

Figure S5. Quantitative analysis of LVEDd, LVEDs, LVEDv, LVESv at 24 h after MIRI according to echocardiography. ***P* < 0.01 versus the MIRI group; ^&^*P* < 0.05 versus the MIRI-MEs-miR-146a group. n=8.

**
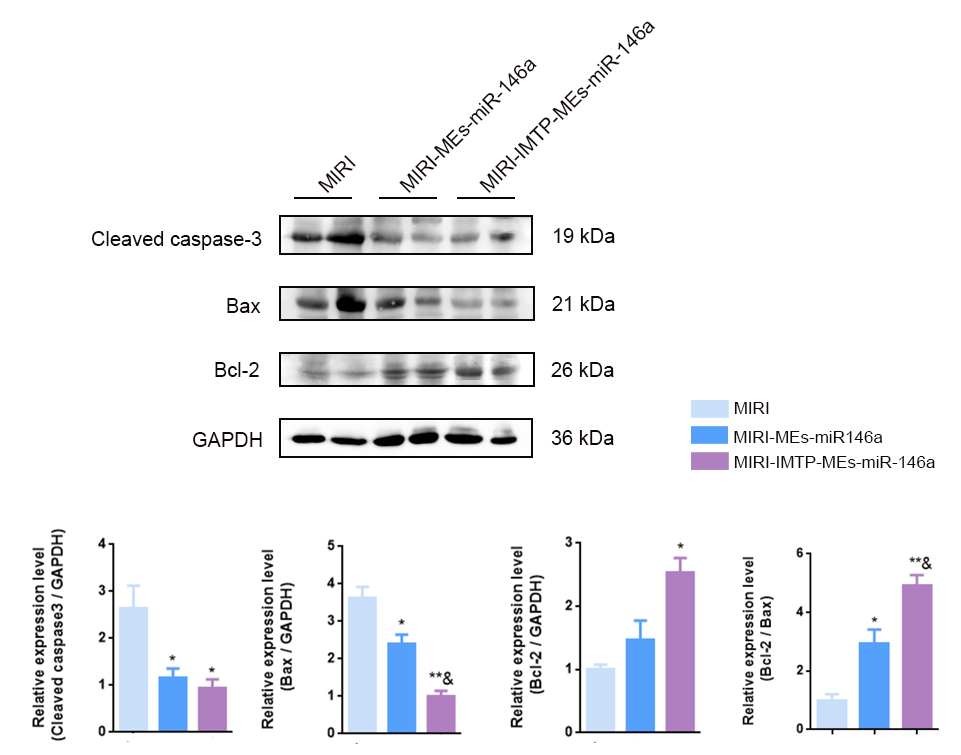
**

Figure S6. Representative western blotting bands and quantification of Cleaved-caspase 3, Bax and Bcl-2 level after intravenous injection of MEs-miR-146a with and without modification. **P* < 0.05, ***P* < 0.01 versus the MIRI group; ^&^*P* < 0.05 versus the MIRI-MEs-miR-146a group. n=4.

**Supplementary Table1. List of antibodies**

| **Antibody** | **Company** | **Catalog number** | **Dilution** |
| --- | --- | --- | --- |
| Anti-rabbit IgG HRP-linked Antibody | Cell Signaling | 7074S | 1:3000 |
| Anti-mouse IgG HRP-linked Antibody | Cell Signaling | 7076S | 1:3000 |
| CD9 | Abcam | ab307085 | 1:1000 |
| CD63 | Santa Cruz Biotechnology | SC-365604 | 1:1000 |
| CD81 | Abcam | ab109201 | 1:1000 |
| TSG101 | Santa Cruz Biotechnology | SC-7964 | 1:1000 |
| IRAK1 | Proteintech | 10478-2-AP | 1:1000 |
| TRAF6 | Cell Signaling | 67591S | 1:1000 |
| p-IκBα | Cell Signaling | 2859S | 1:1000 |
| IκBα | Cell Signaling | 4812S | 1:1000 |
| p-NF-κB p65 | Cell Signaling | 3033S | 1:1000 |
| NF-κB p65 | Cell Signaling | 8242S | 1:1000 |
| Histone H3 | Proteintech | 68345-1-Ig | 1:8000 |
| Cleaved Caspase-3 | Cell Signaling | 9664S | 1:1000 |
| Bax | Cell Signaling | 2772T | 1:1000 |
| Bcl-2 | Abcam | ab196495 | 1:1000 |
| GAPDH | Proteintech | HRP-60004 | 1:8000 |

**Supplementary Table2. List of primers for qRT-PCR**

| **Rat miR-146a** | |  |
| --- | --- | --- |
| Forward | 5’- TGAGAACTGAATTCCATGGGTT-3’ |  |
| Reverse | 5’- TGAGAACTGAATTCCATAGGCTG -3’ |  |
| **Rat U6** | |  |
| Primer Sequence | 5’- TTGGAACGATACAGAGAAGATTAGCAT-3’ |  |
|  |  |  |
| **Rat IRAK1** | |  |
| Forward | 5’- GCAAGTGGGTCTGTTTCTATGG -3’ |  |
| Reverse | 5’- TCTTTCTCTTGGGGTTCTAGGG -3’ |  |
| **Rat TRAF6** | |  |
| Forward | 5’- CAA GTG CCC AGT TGA CAA TG -3’ |  |
| Reverse | 5’- CAC ACA GCC TTT ATT TGG ACA -3’ |  |
| **Rat p65** | |  |
| Forward | 5’-GCT GTT TGG TTT GAG ACA TC-3’ |  |
| Reverse | 5’- TCT GCC CTC CTG ACT CTA CT -3’ |  |
| **Rat GAPDH** | |  |
| Forward | 5’- CATCAAGAAGGTGGTGAAGCA -3’ |  |
| Reverse | 5’- AAGTCACAGGAGACAACCTGGTC -3’ |  |
